# Solitons mediated by skin-mode localization and band nonreciprocity

**DOI:** 10.1093/nsr/nwag381

**Published:** 2026-06-19

**Authors:** Shanyue Li, Mengying Hu, Jing Lin, Chen Fang, Zhensheng Tao, Kun Ding

**Affiliations:** Department of Physics, State Key Laboratory of Surface Physics, and Key Laboratory of Micro and Nano Photonic Structures, Fudan University, Shanghai 200438, China; Department of Physics, State Key Laboratory of Surface Physics, and Key Laboratory of Micro and Nano Photonic Structures, Fudan University, Shanghai 200438, China; Department of Physics, State Key Laboratory of Surface Physics, and Key Laboratory of Micro and Nano Photonic Structures, Fudan University, Shanghai 200438, China; Beijing National Laboratory for Condensed Matter Physics, and Institute of Physics, Chinese Academy of Sciences, Beijing 100190, China; Department of Physics, State Key Laboratory of Surface Physics, and Key Laboratory of Micro and Nano Photonic Structures, Fudan University, Shanghai 200438, China; Department of Physics, State Key Laboratory of Surface Physics, and Key Laboratory of Micro and Nano Photonic Structures, Fudan University, Shanghai 200438, China

**Keywords:** non-Hermitian skin effect, skin-mode-assisted solitons, nonreciprocity-dressed solitons, nonreciprocity, Wannier-projected effective nonlinear Hamiltonian

## Abstract

Solitons typically arise from the competition between band dispersion and nonlinearity. In lattices exhibiting the non-Hermitian skin effect, increasing nonlinearity induces a transition in localization from linear skin modes to solitons. However, localization alone does not clarify how skin modes and band dispersion contribute to soliton formation or classification. Here, by formulating a Wannier-projected effective nonlinear Hamiltonian, we show that soliton formation is determined by how skin-mode localization and band nonreciprocity suppress or enhance wave dispersion, giving rise to two distinct families: skin-mode-assisted solitons (SMASs) and nonreciprocity-dressed solitons (NRDSs). Using a stacked Su–Schrieffer–Heeger-like model as a prototype, we construct the soliton threshold diagram and analytically demonstrate that increasing nonreciprocity lowers the SMAS threshold but raises that of NRDSs. We further extend the framework to higher dimensions and more complex lattices, establishing its generality. This work provides predictive tools for understanding and engineering solitons in experimentally realizable non-Hermitian systems.

## Teaser

This study reveals how skin-mode localization and nonreciprocity shape contrasting routes to soliton formation, offering a general guide for designing nonlinear localized states across dimensions and lattice geometries in non-Hermitian systems.

## INTRODUCTION

Nonlinear wave phenomena are fundamental to diverse physical systems, with solitons as robust and self-localized wave packets, playing a pivotal role in applications such as information transport, nonlinear optics, and signal processing [1–4]. Remarkable advances have been made in harnessing solitons within discrete lattice systems, uncovering myriad phenomena where soliton formation and propagation stem from the interplay between nonlinear self-focusing, band dispersion, and topology [5–15]. The advent of non-Hermitian lattices has revolutionized band dispersion by introducing complex energy, leading to band nonreciprocity under periodic boundary conditions [16–22]. The ensuing topologically nontrivial point gap results in skin-mode localization, known as the non-Hermitian skin effect (NHSE), indicating that eigenstates accumulate exponentially at system boundaries under open boundary conditions in the linear regime [23–34]. When interacting with nonlinearity, the NHSE inherently introduces unprecedented factors into the soliton landscape, offering possibilities that are fundamentally inaccessible in Hermitian settings [35–44].

Recent studies show that, under single-channel excitation, increasing nonlinearity can transform skin modes into trapped solitons, with the excitation site strongly influencing soliton localization [35–37]. This transformation has been experimentally verified [38]. Existing works have adopted a classification scheme based solely on skin-mode localization, employing terms such as edge and bulk skin solitons [36–38], and have focused on systems without sublattices [35–37]. As lattice intricacy increases, identifying various solitons and determining their thresholds theoretically become less straightforward, even in Hermitian systems [4]. NHSE makes it even more complex because higher-dimensional NHSE is sensitive to geometry [45–48] and other factors, not solely to nonreciprocity [18–20]. It is then crucial to quantify the relation between NHSE and solitons, including clarification of soliton formation mechanisms and subsequently predicting soliton thresholds, in a clean-cut manner.

**Figure 1.**
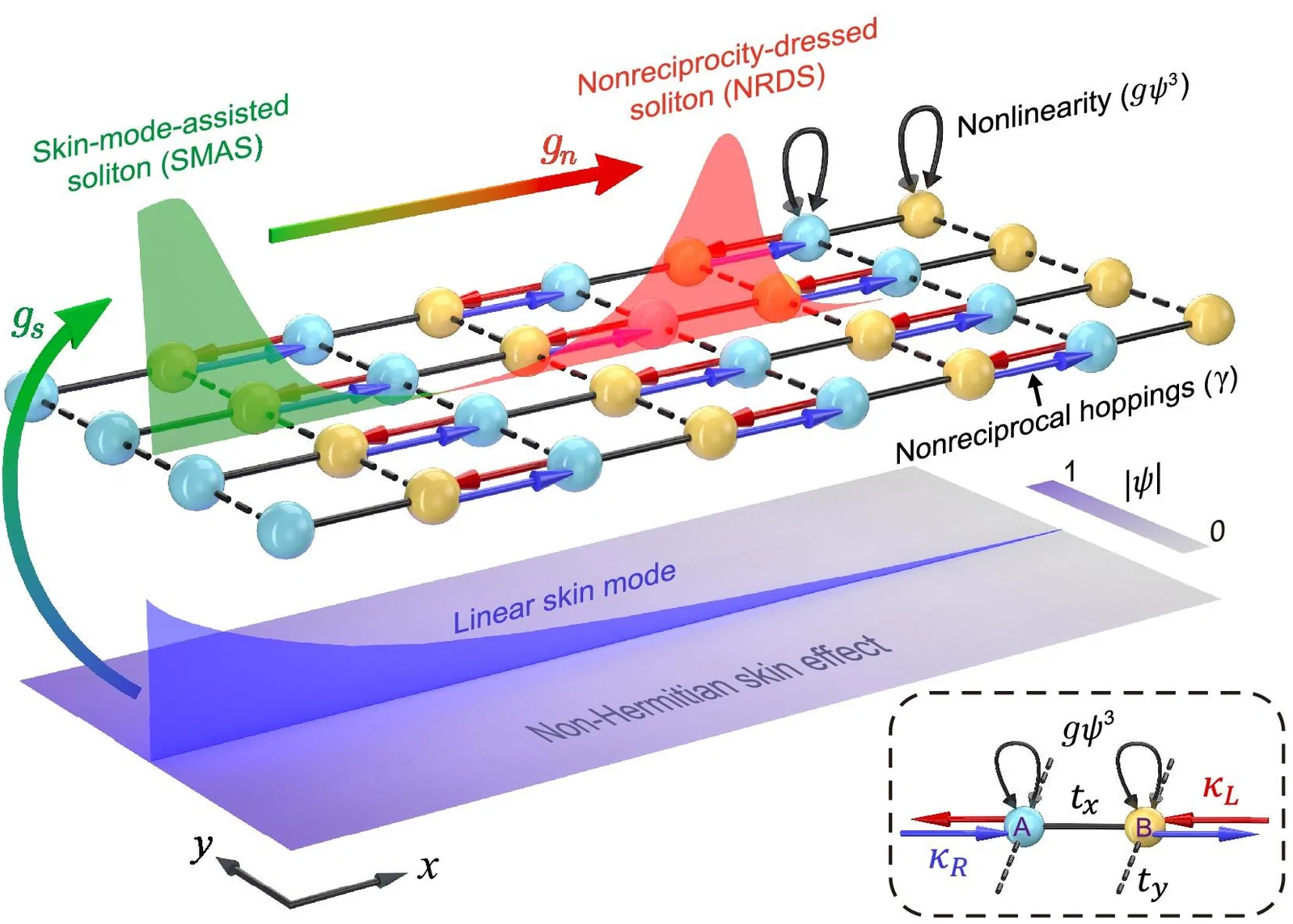
Schematic illustration of skin modes and various solitons. The upper panel shows a two-dimensional lattice featuring nonreciprocal hopping and on-site Kerr nonlinearity, with their respective strengths characterized by *γ * and *g*. Two arrows with gradient color sketch the sequential occurrences from skin modes (blue) to SMASs (green) and subsequently to NRDSs (red). The bottom-right inset displays the unit cell. The intra-cell and *y*-direction inter-cell hoppings are reciprocal, denoted by ${t}_x$ and ${t}_y$, respectively. The *x*-direction inter-cell hopping is nonreciprocal, denoted by ${\kappa }_R$ (rightward) and ${\kappa }_L$ (leftward).

Building on this foundation, we employ a stacked Su–Schrieffer–Heeger-like model as a prototype system that incorporates both nonreciprocal hopping and third-order Kerr nonlinearity (Fig. 1, top). By mapping out the nonlinear threshold diagram, we reveal two distinct soliton families with clearly separated thresholds: skin-mode-assisted solitons (SMASs, green profile in Fig. 1) and nonreciprocity-dressed solitons (NRDSs, red profile in Fig. 1). To quantitatively identify their thresholds and formation mechanisms, we construct a Wannier-projected effective nonlinear Hamiltonian. By retaining dominant Wannier functions to work out the analytical solutions, we demonstrate that the nonlinearity threshold ${g}_s$ (${g}_n$) for SMASs (NRDSs) is governed by the degree to which skin-mode localization (band nonreciprocity) suppresses or enhances linear wave dispersion. With such a framework, we further demonstrate that soliton classifications in nonreciprocal lattices, independent of dimension and lattice types, are determined by skin-mode localizations rather than geometry.

## SKIN-MODE-ASSISTED SOLITONS AND NONRECIPROCITY-DRESSED SOLITONS

To inspect the occurrence of SMASs and NRDSs, we consider a finite lattice under open boundaries (Fig. 1), with the unit cell shown in the inset. The system features nonreciprocal inter-cell hoppings ${\kappa }_R = \kappa ( {1 - \gamma } )$ and ${\kappa }_L = \kappa ( {1 + \gamma } )$, where $\gamma \in [ {0,1} ]$. Nonlinearity is of the Kerr type with strength *g*. For simplicity, we discuss *g < 0*, which corresponds to the focusing regime for *κ < 0*, but our theory also applies when *g > 0*. The real-space eigenvalue equation takes the form


(1) \begin{eqnarray*}
E{\psi }_{\boldsymbol{n}} = {H}_{{\boldsymbol{nm}}}{\psi }_{\boldsymbol{m}} + g{\left| {{\psi }_{\boldsymbol{n}}} \right|}^2{\psi }_{\boldsymbol{n}},
\end{eqnarray*}

where ${\psi }_{\boldsymbol{n}}$ is the wavefunction at site ${\boldsymbol{n}} = ( {{n}_x,{n}_y} )$, with ${n}_x = 1, 2, \cdots ,{N}_x$ and ${n}_y = 1, 2, \cdots ,{N}_y$. ${H}_{{\boldsymbol{nm}}}$ is the linear Hamiltonian and *E* denotes the nonlinear eigenenergy. The number of unit cells along the *x* and *y* directions is ${N}_x/2$ and ${N}_y$, respectively, with odd (even) ${n}_x$ corresponding to sublattice A (B).

**Figure 2.**
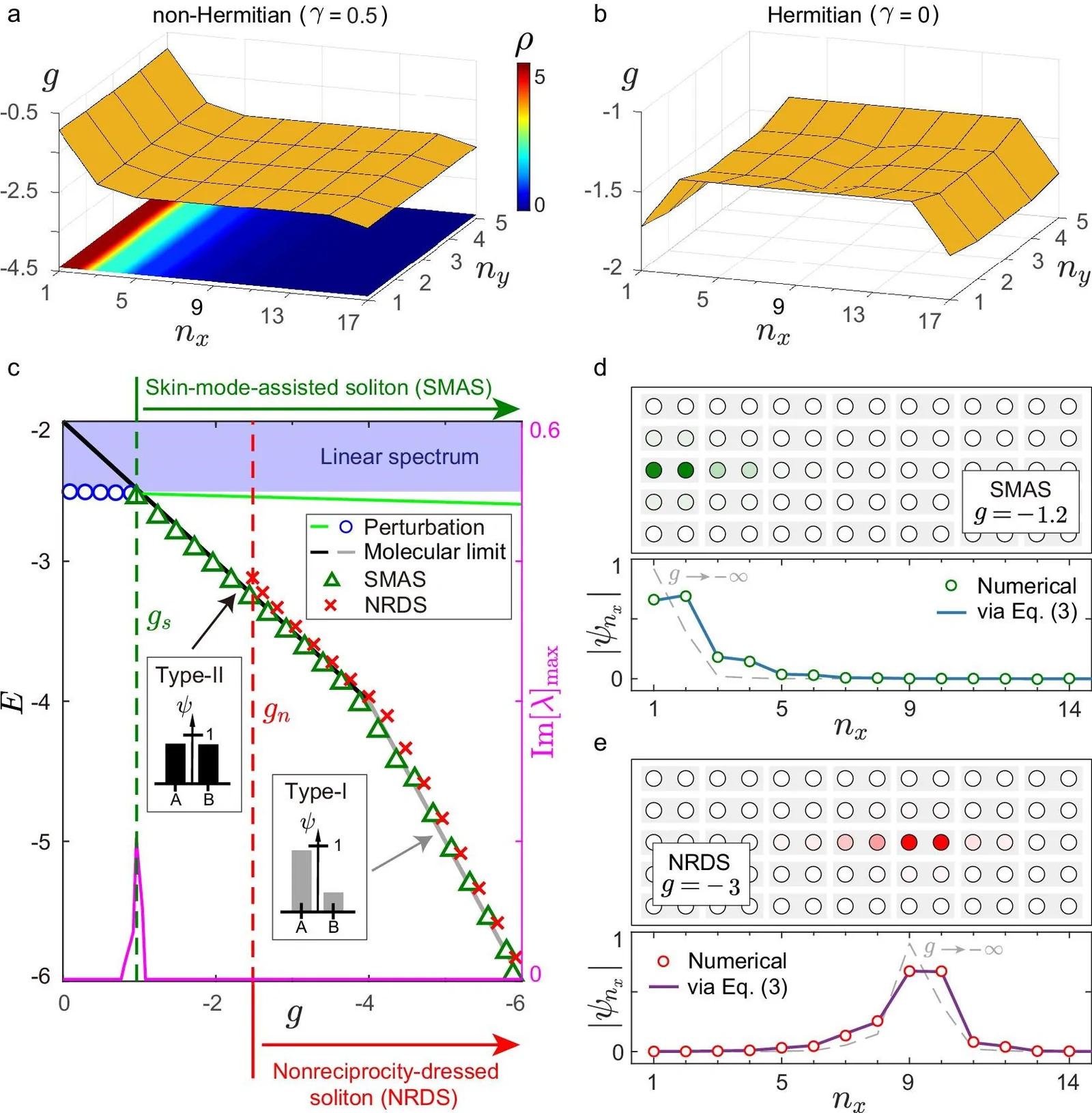
(a and b) Soliton threshold distributions across the lattices in the nonreciprocal (*γ = 0.5*, a) and reciprocal (*γ = 0*, b) cases. Color map in (a) shows the total probability density $\rho = \mathop \sum \nolimits_l {| {{\psi }_l} |}^2$, where ${\psi }_l$ is the *l*th linear skin mode. (c) Spectra of SMASs (green triangles) and NRDSs (red cross markers). The dashed lines mark their respective nonlinear thresholds, ${g}_s = - 0.96$ (green) and ${g}_n = - 2.48$ (red). The black and gray lines indicate analytical spectra in the molecular limit, with insets showing the corresponding eigenstate profiles. The solid green line and blue circles are nonlinear perturbative skin modes obtained from perturbation theory and numerical calculations. The magenta line exhibits ${\mathrm{Im}}{[ \lambda ]}_{{\mathrm{max}}}$, the largest imaginary part of the eigenvalues of the stability matrix for the considered modes. $| {{\psi }_{\boldsymbol{n}}} |$ for an SMAS at *g = - 1.2* (d, upper panel) and an NRDS at *g = - 3* (e, upper panel). The color shading represents the amplitude $| {{\psi }_{\boldsymbol{n}}} |$. The open circles in the lower panels of (d) and (e) show $| {{\psi }_{( {{n}_x,{n}_y = 3} )}} |$ of corresponding solitons in the upper panels, while solid lines are calculated from the effective nonlinear Hamiltonian [Eq. (3)]. The dashed gray lines represent solitons in the anticontinuum limit. All results are obtained in open boundaries, with parameters *κ = - 0.5*, *γ = 0.5*, ${t}_x = 4\kappa $, ${t}_y = 0.1\kappa $, ${N}_x = 18$, and ${N}_y = 5$.

Before solving Eq. (1) in full, we first examine two limiting cases: the linear limit and the molecular limit. Setting *g = 0* recovers the linear regime, where NHSE necessarily arises when $\gamma \not= 0$. The color map in Fig. 2a and the shaded blue region in Fig. 2c, respectively, indicate skin-mode localization and spectral range of the lower-energy band for *γ = 0.5* (see Sec. I of the online Supplementary Material). The molecular limit corresponds to the case with no inter-cell hoppings, i.e., ${\kappa }_R = {\kappa }_L = {t}_y = 0$ [49]. In this regime, Eq. (1) decouples into a series of identical nonlinear equation systems, each consisting of two coupled equations. Solving these decoupled systems and imposing the linearly stable, steady-state condition yields the spectra depicted by the solid black and grey lines in Fig. 2c, with corresponding eigenstate profiles shown in the inset (see Sec. II of the online Supplementary Material). Owing to the underlying sublattices, two distinct types of nonlinear eigenstates emerge: type-I states, which are localized on a single site, and type-II states, which are symmetrically localized across two sites.

With the above preparation, we now numerically solve Eq. (1) using an iterative method (see Sec. III of the online Supplementary Material). To identify and classify solitons, we use molecular-limit eigenstates as initial guesses and systematically record steady states (${\mathrm{Im}}[ E ] = 0$) throughout the entire lattice. Note that we choose to fix the norm of wavefunctions, $\mathop \sum \nolimits_{\boldsymbol{n}} {| {{\psi }_{\boldsymbol{n}}} |}^2 = 1$, and vary *g*, which is equivalent to varying power by fixing *g*. Using this notation, the spatial dependence of soliton thresholds for *γ = 0.5* is shown in Fig. 2a. Soliton thresholds in the skin-mode-localized (nonlocalized) regions are significantly suppressed (boosted), clearly correlating with the skin-mode localization landscape. This is in sharp contrast to the Hermitian case, where soliton thresholds are sensitive to geometric bulk and boundary (Fig. 2b). Thus, Fig. 2c–e displays spectra and mode profiles for representative nonlinear eigenstates both below the soliton threshold and, above thresholds, in skin-mode-localized and nonlocalized regions.

Before solitons emerge at any lattice sites (*g → 0*), nonlinear eigenstates resemble the mode profiles of linear skin modes (see Sec. IV of the online Supplementary Material). Their typical spectra, shown by blue circles in Fig. 2c, match those from regular perturbation theory (solid green line) [39], validating their interpretation as nonlinear perturbative skin modes.

As *g* increases, the intersection of the green and black lines in Fig. 2c hints that nonlinearity is no longer a perturbation and solitons are beginning to emerge. As indicated in Fig. 2a, solitons first emerge near the left boundary, coinciding with the skin-mode localization. Representative spectra (open triangles, Fig. 2c) follow the molecular-limit lines, and their profiles (Fig. 2d) show confinement within a single unit cell, with exponentially decaying tails and a finite boundary amplitude. These solitons emerge from the skin-mode localization and are thus classified as SMASs. We thereby define the critical value *g* for the onset of SMASs within the skin-mode-localized regions as their threshold ${g}_s$ (green dashed line, Fig. 2c).

Away from skin-mode-localized regions, solitons also form but require larger nonlinearity, as evidenced by their spectra (red crosses, Fig. 2c). Their mode profiles (Fig. 2e) show exponential decay on both sides, with decay lengths differing due to nonreciprocal hopping. The profile is irrelevant to the skin boundary and asymmetric, reflecting that its dominant factor is nonreciprocity rather than skin-mode localization. We designate these as NRDSs, with their formation threshold denoted as ${g}_n$ (red dashed line, Fig. 2c).

Both SMAS and NRDS mode profiles in Fig. 2d and e show that, from the symmetry viewpoint, they retain the same types as those in the molecular limit (see insets of Fig. 2c), reflecting sublattice structures. As the nonlinearity ramps up, both SMASs and NRDSs finally evolve into type-I solitons (dashed gray lines, Fig. 2d and e), which connect to the anticontinuum limit (see Sec. V of the online Supplementary Material). Since our focus is on linearly stable and steady-state solitons, we perform a linear stability analysis using the eigenvalues λ of the stability matrix to verify stability (see Sec. VI of the online Supplementary Material) [50]. A nonzero maximum ${\mathrm{Im}}[ \lambda ]$ indicates instability. As plotted by the magenta line in Fig. 2c, nonlinear eigenstates, besides the states near ${g}_s$, are all linearly stable, confirming that both SMASs and NRDSs are stable steady-state solitons sensitive to NHSE. Therefore, to reveal their underlying mechanism, we next examine how ${g}_s$ and ${g}_n$ depend on *γ *.

## THRESHOLD DIAGRAM

**Figure 3.**
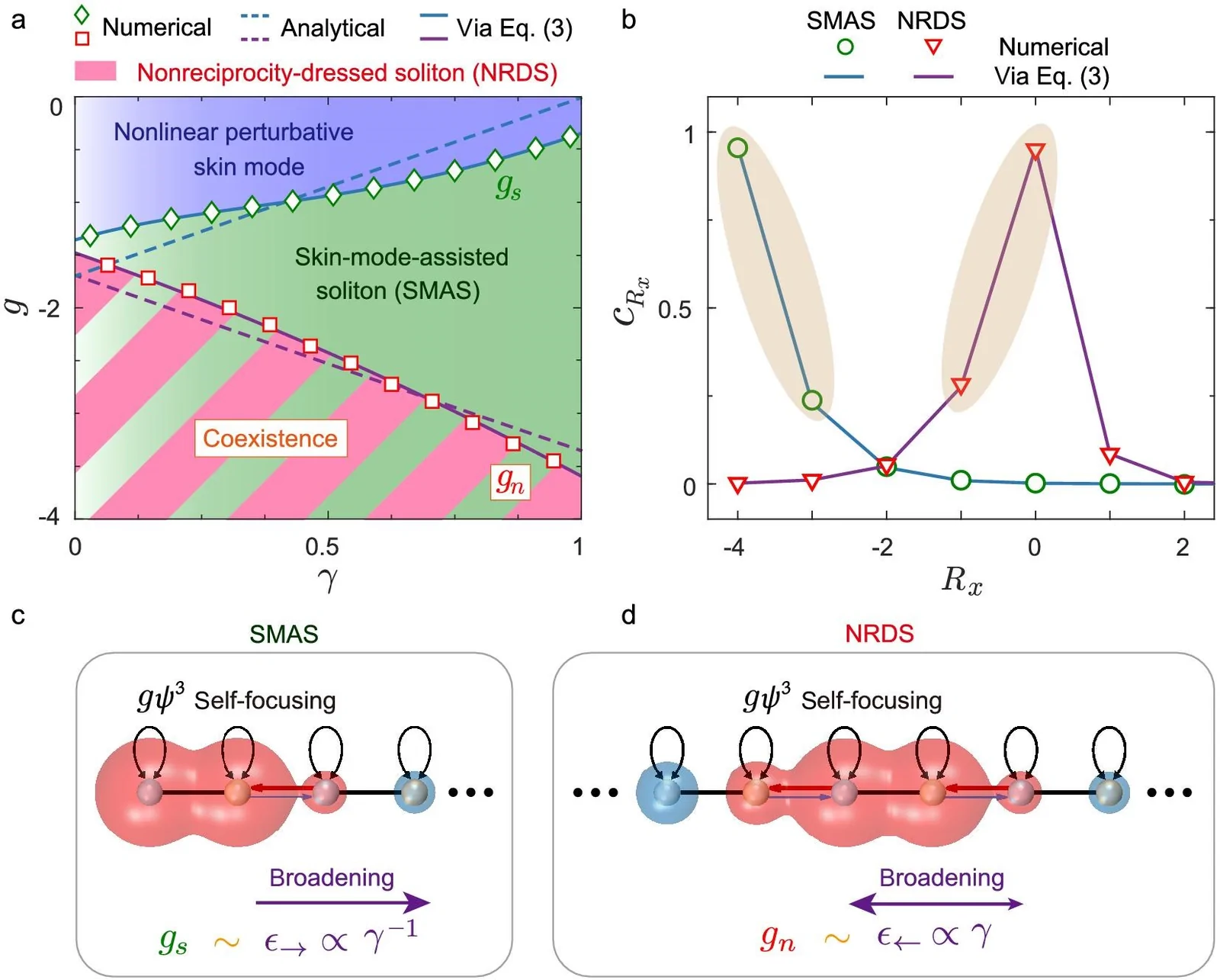
(a) Soliton threshold diagram in the $\gamma {-} g$ plane. Each soliton family is labeled accordingly, with the nonlinear thresholds ${g}_s$ and ${g}_n$ from Eq. (1) indicated by open markers. Solid lines exhibit ${g}_s$ and ${g}_n$ determined using the effective nonlinear Hamiltonian [Eq. (3)], while dashed lines show the analytical results of Eq. (5). (b) Expansion coefficients ${c}_{( {{R}_x,{R}_y = 0} )}$ for the SMAS and NRDS shown in Fig. 2. Open markers (solid lines) are obtained by numerical projection of solitons from Eq. (1) onto the Wannier basis (effective nonlinear Hamiltonian). Shadows mark two leading Wannier functions for each soliton. Schematics showing the formation mechanism of SMASs (c) and NRDSs (d). The red (positive) and blue (negative) surfaces illustrate the isosurface of Wannier functions, centered at the skin-mode-localized sites (c) and non-localized sites (d). The purple arrows denote dominant broadening directions, which are encoded in the molecular hoppings ${\epsilon }_{\rightarrow} \propto {\gamma }^{ - 1}$ and ${\epsilon }_ \leftarrow \propto \gamma $ in the Wannier basis.

We repeat the above analysis for various *γ * and display the threshold diagram in Fig. 3a. Nonlinear perturbative skin modes and two families of solitons under linearly stable and steady-state conditions are mutually exclusive, establishing ${g}_s$ as a critical parameter that separates skin-mode-dominated states from various solitons. SMASs solely exist over a wide range of nonlinearity, i.e., $| {{g}_s} | < | g | < | {{g}_n} |$, while SMASs and NRDSs coexist when $| g | > | {{g}_n} |$. When *γ = 0*, the system reverts to Hermitian behavior: nonlinear perturbative skin modes become delocalized modes, and solitons follow geometric classifications (bulk, edge, and corner).

The distinct *γ *-dependence of ${g}_s$ and ${g}_n$ (open makers) in Fig. 3a indicates different formation mechanisms for two soliton families. Although both SMASs and NRDSs are localized in real space, the onset of SMASs, from a threshold perspective, becomes increasingly easier relative to NRDSs as nonreciprocity grows. This trend must be encoded in their formation mechanisms, motivating a systematic identification of the dominant ingredients determining ${g}_s$ and ${g}_n$.

## REVEALING FORMATION MECHANISMS VIA WANNIER-FUNCTION ANALYSIS

Noting that mode profiles of both SMASs and NRDSs, upon their initial emergence, resemble Wannier functions of the bands from which they bifurcate (see Sec. VII of the Supplementary Material), we construct a nonlinear Hamiltonian in the Wannier basis by expanding the solutions of Eq. (1) as [14,15,51]


(2) \begin{eqnarray*}
\begin{array}{*{20}{c}} {{\psi }_{\boldsymbol{n}} = \mathop \sum \limits_{{\boldsymbol{R}},\alpha } {c}_{{\boldsymbol{R}},\alpha }w_{{\boldsymbol{R}},\alpha ,{\boldsymbol{n}}}^{\mathrm{R}}} \end{array}\!,
\end{eqnarray*}

where ${c}_{{\boldsymbol{R}},\alpha }$ are expansion coefficients, ${\boldsymbol{R}} = ( {{R}_x,{R}_y} )$ denotes the real-space lattice vector, *α * is the band index, and $w_{{\boldsymbol{R}},\alpha ,{\boldsymbol{n}}}^{\mathrm{R}}$ are the Wannier functions constructed from the right eigenvectors (with superscript ${\mathrm{R}}$; ${\mathrm{L}}$ for left). By substituting Eq. (2) into Eq. (1) and assuming nonlinear interactions are dominated by Wannier functions of the valence band at the same unit cell, we obtain the Wannier-projected effective nonlinear Hamiltonian ${H}_{{\boldsymbol{R}},{{\boldsymbol{R}}}^{\boldsymbol{^{\prime}}}}{c}_{{\boldsymbol{R^{\prime}}}} = E{c}_{\boldsymbol{R}}$,


(3) \begin{eqnarray*}
{H}_{{\boldsymbol{R}},{{\boldsymbol{R}}}^{\boldsymbol{^{\prime}}}} = {\epsilon }_{{\boldsymbol{R}},{{\boldsymbol{R}}}^{\boldsymbol{^{\prime}}}} + gW_{{\boldsymbol{R}},{\boldsymbol{R}},{\boldsymbol{R}},{\boldsymbol{R}}}^{\alpha ,\alpha ,\alpha ,\alpha }{\left| {{c}_{\boldsymbol{R}}} \right|}^2{\delta }_{{\boldsymbol{R}},{{\boldsymbol{R}}}^{\boldsymbol{^{\prime}}}},\end{eqnarray*}

where $W_{{\boldsymbol{R}},{\boldsymbol{R}},{\boldsymbol{R}},{\boldsymbol{R}}}^{\alpha ,\alpha ,\alpha ,\alpha }$ is the four-function overlap integral


(4) \begin{eqnarray*}W_{{\boldsymbol{R}},{\boldsymbol{R}},{\boldsymbol{R}},{\boldsymbol{R}}}^{\alpha ,\alpha ,\alpha ,\alpha } = \mathop \sum \limits_{\boldsymbol{n}} {\left( {w_{{\boldsymbol{R}},\alpha ,{\boldsymbol{n}}}^{\mathrm{R}}} \right)}^*w_{{\boldsymbol{R}},\alpha ,{\boldsymbol{n}}}^{\mathrm{L}}w_{{\boldsymbol{R}},\alpha ,{\boldsymbol{n}}}^{\mathrm{R}}w_{{\boldsymbol{R}},\alpha ,{\boldsymbol{n}}}^{\mathrm{R}},\end{eqnarray*}

with *α * taken as the valence band. ${\epsilon }_{{\boldsymbol{R}},{{\boldsymbol{R}}}^{\boldsymbol{^{\prime}}}}$ are the hoppings in the Wannier basis. Despite its simplifying assumptions, the effective nonlinear Hamiltonian [Eq. (3)] reproduces both SMASs and NRDSs with high accuracy, as shown by the solid lines in Fig. 2d and e. Accordingly, ${g}_s$ and ${g}_n$ in the threshold diagram are successfully reproduced (solid lines, Fig. 3a), highlighting the Wannier-function approach as a powerful tool for elucidating the underlying mechanisms of solitons.

Figure 3b compares two sets of expansion coefficients ${c}_{\boldsymbol{R}}$: one obtained from Eq. (3) and plotted as solid lines, the other derived by projecting the numerically calculated solitons (upper panels in Fig. 2d and e) onto the Wannier basis and displayed as open markers. As both SMASs and NRDSs are dominated by only a few Wannier functions, we retain the two leading ones (marked by shadows) and work out ${g}_s$ and ${g}_n$ analytically (see Sec. VII of the online Supplementary Material). This reduces the nonlinear Hamiltonian of Eq. (3) from an *N*-by-*N* matrix to a 2-by-2 one, where *N* is the total number of unit cells. Imposing steady-state conditions yields the analytical thresholds (dashed lines, Fig. 3a)


(5) \begin{eqnarray*}{g}_s = \sqrt {\frac{{11 + 5\sqrt{5} }}{2}} \frac{{{\epsilon }_ \to }}{W},\ \ {g}_n = \sqrt {\frac{{11 + 5\sqrt{5} }}{2}} \frac{{{\epsilon }_ \leftarrow }}{W},
\end{eqnarray*}

where $W = W_{{\boldsymbol{R}},{\boldsymbol{R}},{\boldsymbol{R}},{\boldsymbol{R}}}^{\alpha ,\alpha ,\alpha ,\alpha }$ is the dominant nonlinear interaction, and ${\epsilon }_ \to $ and ${\epsilon }_ \leftarrow $ are the rightward and leftward nearest-neighbor hoppings in the Wannier basis (in the molecule sense). Nonreciprocity implies ${\epsilon }_ \leftarrow $$\propto \gamma $, while ${\epsilon }_ \to \propto {\gamma }^{ - 1}$. Recalling that solitons result from the balance between linear dispersion (broadening) and nonlinearity self-focusing, Eq. (5) implies distinct formation mechanisms for SMASs and NRDSs, as detailed below.

Figure 3c and d shows representative Wannier functions in skin-mode-localized and nonlocalized regions, and illustrates their dominant broadening tendencies. For solitons at skin-mode-localized regions, skin-mode localization suppresses broadening in both directions, leading to a decrease in the SMAS threshold ${g}_s$ with stronger *γ * (Fig. 3a and c). In contrast, for solitons at non-localized regions, band nonreciprocity enhances broadening by driving the waves directionally while simultaneously promoting diffusion, thereby increasing the NRDS threshold ${g}_n$ as *γ * grows (Fig. 3a and d). This analysis clarifies the distinct roles of skin-mode localization and band nonreciprocity in soliton formation, justifying the terminology of SMASs and NRDSs. We adopt these two phrases because we deem that skin-mode localization renders soliton classification by the boundaries and bulk inappropriate. With such a framework established, we now generalize to incorporate dimensionality and lattice types, as discussed below.

## EXTENDED VALIDITY ACROSS DIMENSIONS

**Figure 4.**
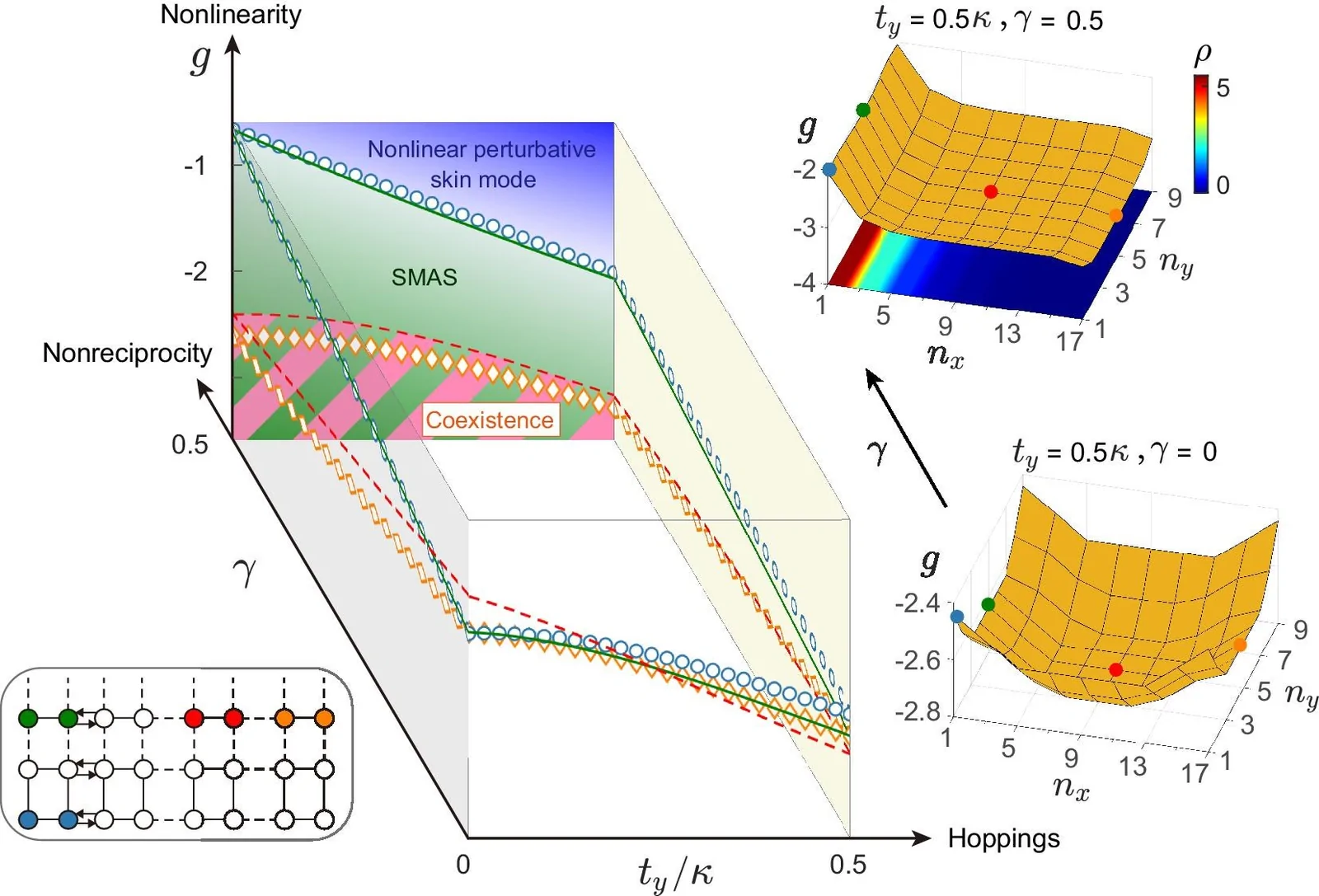
The left panel displays soliton thresholds (*g*) varying with non-Hermiticity (*γ *) and dimensionality (tuned by ${t}_y$). The solid green lines, diamonds, circles, and dashed red lines correspond to the thresholds of solitons appearing at the left edge, right edge, bottom-left corner, and the bulk of the lattice (inset), respectively. The right panel displays soliton threshold distributions for one ${t}_y$, representing a 2D scenario. The bottom and top subpanels show the reciprocal (*γ = 0*) and nonreciprocal (*γ = 0.5*) cases. The color map in the top subpanel shows the total probability density *ρ *. All results are obtained in open boundaries with ${N}_y = 9$.

We have shown that NHSE reshapes soliton-threshold landscapes, shifting the determining factor from geometric location in Hermitian systems to skin-mode localization in non-Hermitian lattices. While the previous analysis focuses on one particular ${t}_y$, we now broaden the scope by varying ${t}_y$ to encompass dimensionality. As is known, the soliton threshold in two-dimensional Hermitian lattices increases monotonically from corners to edges and then to the bulk (Fig. 4, bottom-right panel), a behavior that distinctly differs from the one-dimensional limit (Fig. 2b). To map this dimensionality crossover, the front plane of Fig. 4 (left panel) depicts the thresholds of solitons localized at four representative lattice positions (see inset) when ${t}_y$ changes continuously, revealing a critical ${t}_y$ value that separates one-dimensional and two-dimensional soliton behavior.

Non-Hermiticity fundamentally breaks this hierarchy. As clearly shown in Fig. 2a and the top-right panel of Fig. 4, soliton thresholds under NHSE no longer follow geometric or dimensional rules. Instead, they universally adhere to the skin-mode localization landscape, being suppressed in skin-mode-localized regions and enhanced elsewhere, irrespective of dimensionality (see Sec. VIII of the online Supplementary Material). Consequently, with increasing non-Hermiticity, the four thresholds split into two distinct groups in both the one-dimensional (${t}_y = 0$) and two-dimensional (${t}_y = 0.5\kappa $) configurations, as seen in the left and right planes of Fig. 4 (left panel). The dashed red lines and orange diamonds correspond to one group, while the remaining two represent the other. Their evolution with ${t}_y$ is further illustrated in the rear plane. Notably, the dimensionality crossover observed in Hermitian systems disappears, underscoring a fundamental departure from Hermitian behavior. Therefore, the classification of solitons in non-Hermitian lattices must, regardless of dimensionality, adhere to the criterion dictated by skin-mode localization.

## EXTENDED VALIDITY ACROSS LATTICE GEOMETRIES

**Figure 5.**
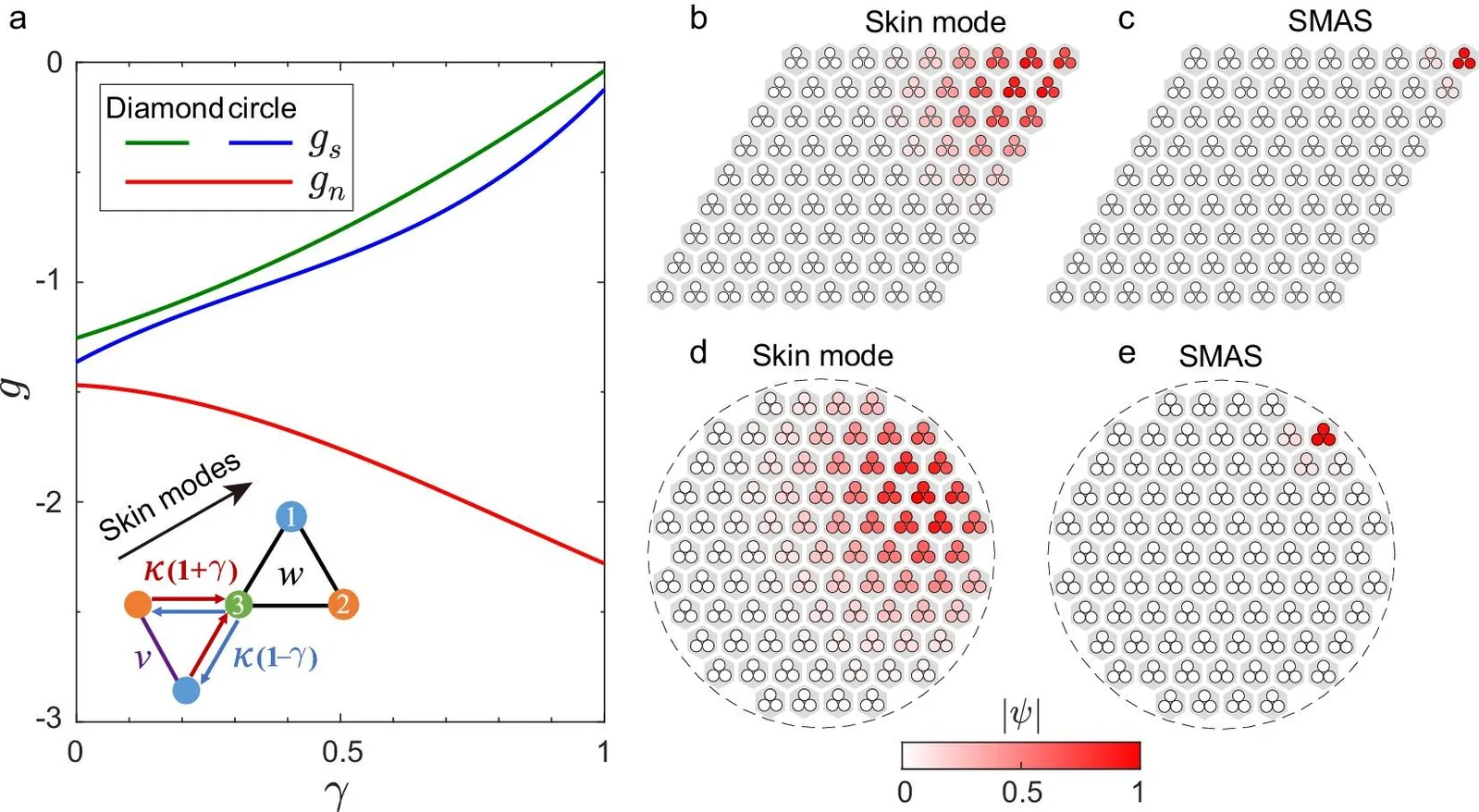
(a) Nonlinear thresholds of SMASs (${g}_s$) and NRDSs (${g}_n$) in nonreciprocal Kagome lattices. The green and blue lines correspond to ${g}_s$ under diamond and circle open boundaries, respectively, while the red line denotes ${g}_n$ for both boundaries. The inset shows the unit cell, where the reciprocal hoppings are labeled as *w* and *v*. The *x*-direction and off-diagonal direction inter-cell hoppings are nonreciprocal, denoted by $\kappa ( {1 + \gamma } )$ and $\kappa ( {1 - \gamma } )$. (b–e) $| {{\psi }_{\boldsymbol{n}}} |$ of skin modes (b and d) and SMASs (c and e) at *γ = 0.5*. In (c and e) or (b and d), *g = - 1.2* or 0. Panels (b and c) and (d and e) correspond to the diamond and circle boundaries, respectively. The color scale in (b–e) represents the maximum-normalized amplitude $| {{\psi }_{\boldsymbol{n}}} |$. The parameters are *κ = - 0.25*, *w = 4κ *, and *v = 0.1κ *.

To further illustrate, we investigate a nonlinear and nonreciprocal Kagome lattice with open boundaries, whose unit cell is shown in the inset of Fig. 5a. We still use the parameter *γ * to tune the nonreciprocity strength. The localization tendency of linear skin modes is indicated by the arrow in the inset. To further verify the effect of boundary shape, we consider two geometries: diamond (Fig. 5b and c) and circle (Fig. 5d and e). The corresponding skin modes localize at the corner and along the preferred boundary, respectively (Fig. 5b and d). We utilize the established framework to diagnose the steady-state solitons therein. As shown in Fig. 5c and e, SMASs also emerge at sites with the skin-mode localization. The corresponding SMAS threshold ${g}_s$ is depicted in Fig. 5a by solid green and blue lines, and follows the same trend as that in the Su–Schrieffer–Heeger-like models shown in Fig. 1, i.e., ${g}_s$ decreases as *γ * ramps up. We also calculate the NRDS threshold ${g}_n$, as shown by the solid red line in Fig. 5a. ${g}_n$ increases against *γ * (closely mirrors that in Fig. 3a), further confirming that NRDS formation is governed by the wave-broadening enhancement induced by band nonreciprocity. Therefore, the same formation mechanisms apply even though the SMASs therein emerge at corners rather than edges, underscoring the ubiquity and robustness of our framework across distinct non-Hermitian lattice geometries.

## DISCUSSIONS AND CONCLUSIONS

All soliton families have been theoretically clarified by isolating the roles of skin-mode localization and band nonreciprocity, and we further examine their dynamical accessibility. Both soliton families can be dynamically excited in their localized regions and subsequently propagate stably (see Sec. IX of the online Supplementary Material). This observation bridges our theoretical framework with physically realizable excitations, confirming the emergence of robust nonlinear eigenstates and distinguishing them from the purely linear NHSE dynamics.

In summary, by clarifying a classification criterion intrinsic to the NHSE, we distinguish two soliton families from numerically obtained nonlinear eigenstates supported in non-Hermitian lattices, dubbed SMAS and NRDS, which respectively owe to skin-mode localization and band nonreciprocity. Using this classification, we construct the soliton threshold diagram as a function of nonreciprocity and dimensionality. To interpret and predict these thresholds, we develop a Wannier-projected effective nonlinear Hamiltonian that captures the threshold values and reveals the dominant mechanisms for each soliton family. The formation of SMAS (NRDS) roots in the suppression or enhancement of wave dispersion from skin-mode localization (band nonreciprocity). Regardless of dimensionality and lattice intricacy, SMASs (NRDSs) emerge at the lattice sites where skin modes are localized (or not), highlighting the generality of our framework. Given the experimental realization of non-Hermitian lattices with nonlinearity [38,40,52,53], our framework provides a practical tool for analyzing the interplay between skin modes and solitons and, potentially, extends to gap solitons and nonlinear topological modes [5–15,40–43]. Furthermore, the anomalous dynamical behaviors of solitons in non-Hermitian lattices [35–38,40,52,53], combined with the stability analysis [54,55], suggest promising avenues for controlling solitons. Our results thus provide both conceptual insight and a strategy for soliton engineering in non-Hermitian systems.

## METHODS

We here describe in detail the construction of the Wannier-projected effective nonlinear Hamiltonian. Substituting Eq. (2) into Eq. (1), multiplying both sides by $w_{{\boldsymbol{R^{\prime}}},\alpha ^{\prime},{\boldsymbol{n}}}^{\mathrm{L}}$, and summing over all lattice sites ${\boldsymbol{n}}$ yields


(6) \begin{eqnarray*}
E{c}_{{\boldsymbol{R^{\prime}}},\alpha ^{\prime}} &=& \mathop \sum \limits_{{\boldsymbol{R}},\alpha } {c}_{{\boldsymbol{R}},\alpha }\mathop \sum \limits_{{\boldsymbol{n}},{\boldsymbol{m}}} w_{{\boldsymbol{R^{\prime}}},\alpha ^{\prime},{\boldsymbol{n}}}^{\mathrm{L}}{H}_{{\boldsymbol{nm}}}w_{{\boldsymbol{R}},\alpha ,{\boldsymbol{m}}}^{\mathrm{R}}\\
&+& \mathop \sum \limits_{\begin{array}{@{}*{1}{c}@{}}\\smallriptstyle {\boldsymbol{R},{\boldsymbol{R^{\prime\prime}}},{\boldsymbol{R^{\prime\prime\prime}}}}\\ {\alpha ,\alpha ^{\prime\prime},\alpha ^{\prime\prime\prime},} \end{array}} gW_{{\boldsymbol{R}},{\boldsymbol{R^{\prime}}},{\boldsymbol{R^{\prime\prime}}},{\boldsymbol{R^{\prime\prime\prime}}}}^{\alpha ,\alpha ^{\prime},\alpha ^{\prime\prime},\alpha ^{\prime\prime\prime}}c_{{\boldsymbol{R}},\alpha }^*{c}_{{\boldsymbol{R^{\prime\prime}}},\alpha ^{\prime\prime}}{c}_{{\boldsymbol{R^{\prime\prime\prime}}},\alpha ^{\prime\prime\prime}},\\
\end{eqnarray*}

where $W_{{\boldsymbol{R}},{\boldsymbol{R^{\prime}}},{\boldsymbol{R^{\prime\prime}}},{\boldsymbol{R^{\prime\prime\prime}}}}^{\alpha ,\alpha ^{\prime},\alpha ^{\prime\prime},\alpha ^{\prime\prime\prime}}$ is the overlap integral of four Wannier functions,


(7) \begin{eqnarray*}
&& W_{{\boldsymbol{R}},{\boldsymbol{R^{\prime}}},{\boldsymbol{R^{\prime\prime}}},{\boldsymbol{R^{\prime\prime\prime}}}}^{\alpha ,\alpha ^{\prime},\alpha ^{\prime\prime},\alpha ^{\prime\prime\prime}}\\
&&\quad\quad = \mathop \sum \limits_{\boldsymbol{n}} {\left( {w_{{\boldsymbol{R}},\alpha ,{\boldsymbol{n}}}^{\mathrm{R}}} \right)}^*w_{{\boldsymbol{R^{\prime}}},\alpha ^{\prime},{\boldsymbol{n}}}^{\mathrm{L}}w_{{\boldsymbol{R^{\prime\prime}}},\alpha ^{\prime\prime},{\boldsymbol{n}}}^{\mathrm{R}}w_{{\boldsymbol{R^{\prime\prime\prime}}},\alpha ^{\prime\prime\prime},{\boldsymbol{n}}}^{\mathrm{R}}.\\
\end{eqnarray*}

To simplify Eq. (6), we transform the real-space linear Hamiltonian ${H}_{{\boldsymbol{nm}}}$ into the Wannier basis, which allows us to rewrite Eq. (6) as


(8) \begin{eqnarray*}
E{c}_{{\boldsymbol{R}},\alpha } &=& \mathop \sum \limits_{{\boldsymbol{R^{\prime}}}} {\epsilon }_{{\boldsymbol{R}},{\boldsymbol{R^{\prime}}},\alpha }{c}_{{\boldsymbol{R^{\prime}}},\alpha }\\
&+& \mathop \sum \limits_{\begin{array}{@{}*{1}{c}@{}}\\smallriptstyle {{\boldsymbol{R^{\prime}}},{\boldsymbol{R^{\prime\prime}}},{\boldsymbol{R^{\prime\prime\prime}}}}\\ {\alpha ^{\prime},\alpha ^{\prime\prime},\alpha ^{\prime\prime\prime},} \end{array}} gW_{{\boldsymbol{R^{\prime}}},{\boldsymbol{R}},{\boldsymbol{R^{\prime\prime}}},{\boldsymbol{R^{\prime\prime\prime}}}}^{\alpha ,\alpha ^{\prime},\alpha ^{\prime\prime},\alpha ^{\prime\prime\prime}}c_{{\boldsymbol{R^{\prime}}},\alpha ^{\prime}}^*{c}_{{\boldsymbol{R^{\prime\prime}}},\alpha ^{\prime\prime}}{c}_{{\boldsymbol{R^{\prime\prime\prime}}},\alpha ^{\prime\prime\prime}},\\
\end{eqnarray*}

where ${\epsilon }_{{\boldsymbol{R}},{\boldsymbol{R^{\prime}}},\alpha }$ are the hoppings in the Wannier basis. We have swapped ${\boldsymbol{R}}$ and ${\boldsymbol{R^{\prime}}}$ (*α * and $\alpha ^{\prime}$) from Eq. (6) to Eq. (8). Since no assumptions have yet been made, Eqs (1) and (8) are thus equally valid for determining static solitons. Their sole distinction lies in the basics: ${\psi }_{\boldsymbol{n}}$ gives the wave amplitude on individual real-space lattice sites, whereas ${c}_{{\boldsymbol{R}},\alpha }$ is the corresponding coefficient in the Wannier basis. To advance the analysis, we make two reasonable simplifications: (i) ignore the nonlinear band-to-band coupling terms in Eq. (8) and consider only a single band *α *. This is because the energy bands of our system are nondegenerate at any momentum ${\boldsymbol{k}}$, and the maximum nonlinear contribution $\max [ {g{{| {{\psi }_{\boldsymbol{n}}} |}}^2} ]$ to Hamiltonian within the *g* of interest is less than the linear band gaps. (ii) Keep diagonal terms in $W_{{\boldsymbol{R^{\prime}}},{\boldsymbol{R}},{\boldsymbol{R^{\prime\prime}}},{\boldsymbol{R^{\prime\prime\prime}}}}^{\alpha ,\alpha ^{\prime},\alpha ^{\prime\prime},\alpha ^{\prime\prime\prime}}$, namely ${\boldsymbol{R}} = {\boldsymbol{R^{\prime}}} = {\boldsymbol{R^{\prime\prime}}} = {\boldsymbol{R^{\prime\prime\prime}}}$. Because our bands are not topological, Wannier functions not only are exponentially attenuated at infinity but also decay immediately away from the Wannier center.

Applying these two approximations, Eq. (8) reduces to the Hamiltonian form given in Eq. (3). Within a periodic-boundary setting, the hopping elements ${\epsilon }_{{\boldsymbol{R}},{{\boldsymbol{R}}}^{\boldsymbol{^{\prime}}}}$ can be obtained by Fourier transforming the Bloch band dispersion ${E}_{\boldsymbol{k}}$, i.e., ${\epsilon }_{{\boldsymbol{R}},{{\boldsymbol{R}}}^{\boldsymbol{^{\prime}}}} = {\epsilon }_{{\boldsymbol{R^{\prime}}} - {\boldsymbol{R\ }}} = [ {\mathop \sum \nolimits_{\boldsymbol{k}} {e}^{ - i{\boldsymbol{k}} \cdot ( {{\boldsymbol{R^{\prime}}} - {\boldsymbol{R}}} )}{E}_{\boldsymbol{k}}} ]/N$. Open boundaries are subsequently imposed by setting the boundary elements in ${\epsilon }_{{\boldsymbol{R^{\prime}}} - {\boldsymbol{R }}}$ to zero.

## Supplementary Material

nwag381_Supplemental_File
