# Supplementary material for: Solitons mediated by skin-mode localization and band nonreciprocity
Source: Natl Sci Rev. 2026 Jun 19;13(17):nwag381. doi: 10.1093/nsr/nwag381 (PMC13548880; doi:10.1093/nsr/nwag381)
Supplement: Supplementary file 1 [file nwag381_supplemental_file.pdf]

Supplementary Material for

Solitons Mediated by Skin-Mode Localization and Band Nonreciprocity

Shanyue Li[1], Mengying Hu[1], Jing Lin[1], Chen Fang[2], Zhensheng Tao[1], and Kun Ding[1]

[1]*Department of Physics, State Key Laboratory of Surface Physics, and Key Laboratory of Micro and Nano Photonic Structures, Fudan University, Shanghai 200438, China*

[2]*Beijing National Laboratory for Condensed Matter Physics, and Institute of Physics, Chinese Academy of Sciences, Beijing 100190, China*

This Supplementary Material contains 9 sections, 14 figures, and 10 references.

CONTENTS

Section I. The linear limit

Section II. The molecular limit

Section III. Numerical method for solving nonlinear equations

Section IV. Regular perturbation theory

Section V. From type-II to type-I solitons

Section VI. Linear stability analysis

Section VII. Wannier-projected effective nonlinear Hamiltonian

Section VIII. Impact of the y direction on soliton formation

Section IX. Dynamical accessibility of solitons

Section I. The linear limit

The Bloch Hamiltonian of the stacked nonreciprocal Su-Schrieffer-Heeger (SSH) model used in the main text is

$$H(\boldsymbol{k}) = \begin{pmatrix} 2t_y \cos k_y & t_x + \kappa_R e^{-ik_x} \\ t_x + \kappa_L e^{ik_x} & 2t_y \cos k_y \end{pmatrix}, \tag{S1.1}$$

where $\boldsymbol{k} = (k_x, k_y)$ is the wave vector of the momentum space. The Hamiltonian has chiral symmetry, i.e., $\sigma_Z H(\boldsymbol{k})\sigma_Z = -H(\boldsymbol{k})$, which allows the system to have paired spectra in the form of $(E, -E)$, as shown in Figs. S1(a) and S1(b). Under periodic boundary conditions, nonreciprocity ($\gamma \neq 0$) makes the spectra (red) change from purely real [Fig. S1(a)] to complex [Fig. S1(b)]. The model can be considered as a collection of periodic nonreciprocal SSH chains with a momentum-dependent chemical potential $2t_y \cos k_y$, such that the spectra consist of a set of ellipses displaced relative to each other along the real-energy axis, supporting two topologically nontrivial point gaps [1,2].

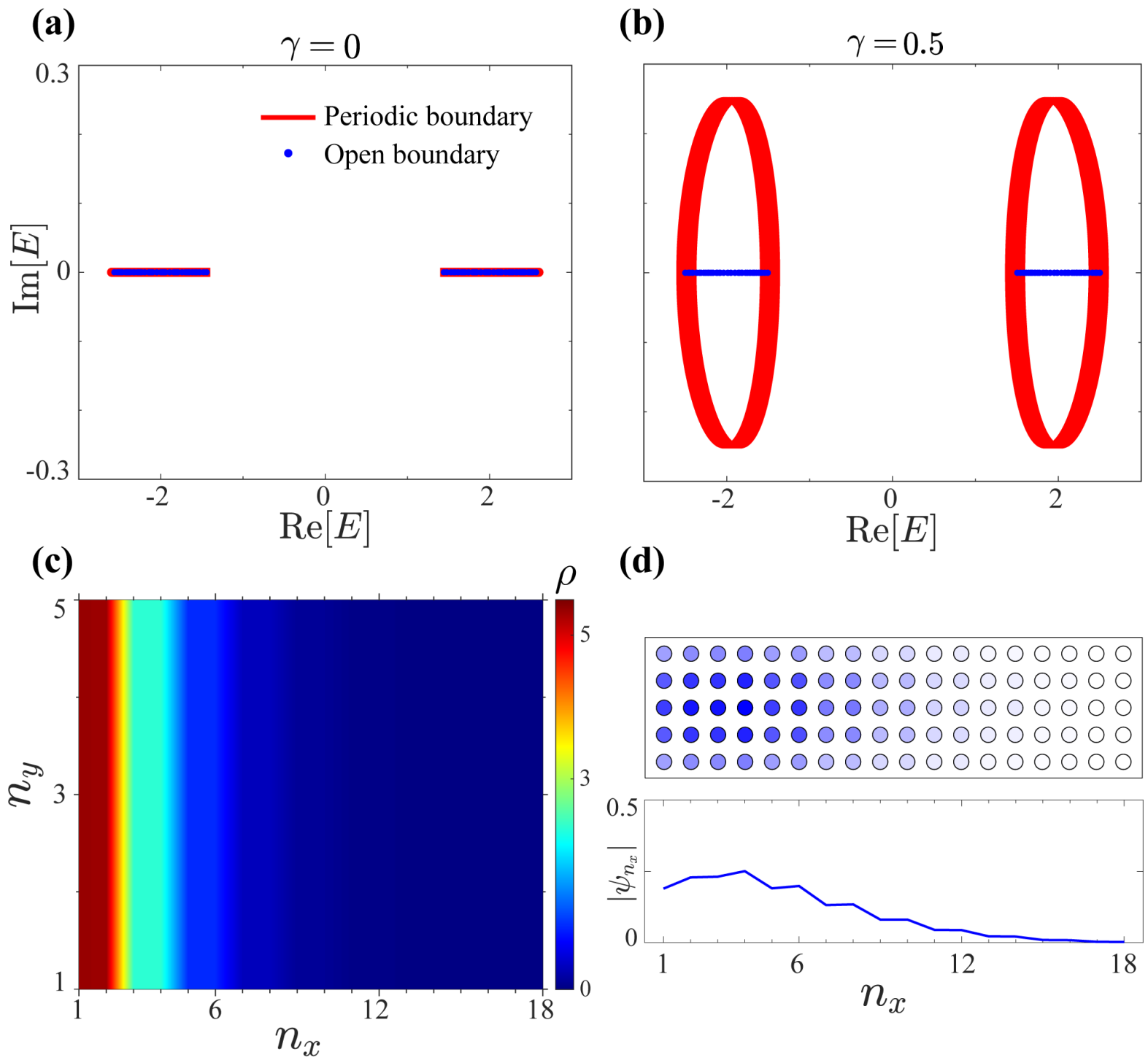

Figure S1. (a) Linear spectra of the stacked nonreciprocal SSH model under periodic boundary conditions (solid red lines) and open boundary conditions (blue dots) in the Hermitian case with $\gamma = 0$. (b) Same as (a) but for the non-Hermitian case with $\gamma = 0.5$. (c) Spatial distribution of the total probability density ρ for the $\gamma = 0.5$ case. (d) Spatial distribution (upper panel) of $|\psi_{\boldsymbol{n}}|$ for the lowest-energy skin mode under open boundary conditions at $\gamma = 0.5$. The lower panel shows $|\psi_{(n_x, n_y=3)}|$ as a function of n_x for this skin mode. Other parameters are identical to those in the main text.

In the Hermitian case, the open-boundary spectra (blue) coincide with the periodic-boundary spectra [Fig. S1(a)]. In the non-Hermitian case, the significant difference between the open-boundary spectra and the periodic-boundary spectra [Fig. S1(b)] leads to the eigenstates under open boundary conditions being significantly different from those of the modulated plane waves under periodic boundary conditions, and are represented by skin modes localized at the favored boundary, which are exponentially attenuated along the x-direction. This indicates that the nonreciprocity along the x-direction leads to the formation of the non-Hermitian skin effect (NHSE). To describe this phenomenon, we consider the real-space probability density summed over all linear skin modes

$$\rho(\boldsymbol{r}) = \sum_{l} |\langle \boldsymbol{r}|\psi_l\rangle|^2 , \tag{S1.2}$$

where $\boldsymbol{r} = (x, y)$ is the position of a lattice site and ψ_l is the l-th linear skin mode. The total probability density ρ for $\gamma = 0.5$ plotted in Fig. S1(c) shows the exponential accumulation of the skin modes along the x-direction toward the left boundary. Specifically, we show in Fig. S1(d) the real-space distribution of the skin mode corresponding to the lowest open-boundary energy. Notably, each nonreciprocal SSH chain in the stack produces its own NHSE and has the same localization length $\xi_x = \frac{1}{-\ln|\beta_x|} a$, where a is the lattice constant and $|\beta_x| = \sqrt{\left|\frac{1-\gamma}{1+\gamma}\right|}$ [3]. Therefore, at $\gamma = 0.5$, the localization length in the x-direction is $\xi_x = 1.82a$ [Fig. S1(c)], which sets the characteristic scale that separates skin-mode-localized regions from effectively non-localized regions. The values of γ can go beyond $[0,1]$, but we focus on the $\gamma \in [0,1]$ situation here, and the generalization to other parameters is straightforward.

Section II. The molecular limit

Solving Eq. (1) in the main text numerically is usually performed iteratively, and the direction in which the iteration progresses and whether it can converge depend on the initial guesses we are given at the beginning of the iteration. Here, we can utilize the molecular limit to obtain exact solutions [4] and use them as a starting point for the nonlinear iterations.

The molecular limit is obtained by ignoring the inter-cell hoppings, as illustrated in Fig. S2(a). In this molecular limit, Eq. (1) in the main text has been simplified as

$$E\psi_A = g\psi_A^3 + t_x\psi_B,$$
$$E\psi_B = g\psi_B^3 + t_x\psi_A. \tag{S2.1}$$

It has been assumed that $\psi_{A/B} \in \mathbb{R}$ due to time-reversal symmetry [4], and the $g|\psi_{A/B}|^2\psi_{A/B}$ term becomes $g\psi_{A/B}^3$. Solving Eq. (S2.1), we can obtain two types of nonlinear eigenstates. For type-I states, the normalized solutions are in the form of the following

$$\psi^{(\mathrm{I},\pm)} = \begin{pmatrix} \psi_A \\ \psi_B \end{pmatrix} = \begin{pmatrix} x_\pm \\ x_\mp \end{pmatrix}, \qquad E = g, \tag{S2.2}$$

where $x_\pm = \pm\sqrt{\left(1 \pm \sqrt{1 - 4t_x^2/g^2}\right)/2}$ and $x_\mp = t_x/(gx_\pm)$. The solid gray lines in Fig. S2(b) show the spectra, and their eigenstate profiles are depicted on the right. Since we have already set $\psi_{A/B} \in \mathbb{R}$, $x_\pm \in \mathbb{R}$ is required, and thus, $g \leq 2t_x$ or $g \geq -2t_x$ (with $t_x < 0$) should be satisfied. The dashed gray line marks the failure of such a requirement, indicating the absence of steady-state solutions. Hence, for $g < 0$, type-I states exist only when $g \leq 2t_x$. To test whether this conclusion extends beyond the molecular limit, we perform numerical calculations for type-I solitons with $g < 0$ in the lattice under realistic hopping configurations. The results show that no steady-state solutions exist for g larger than a critical value g_t, which lies close to $g = 2t_x$. This confirms that type-I solitons inherit the properties of type-I states in the molecular limit.

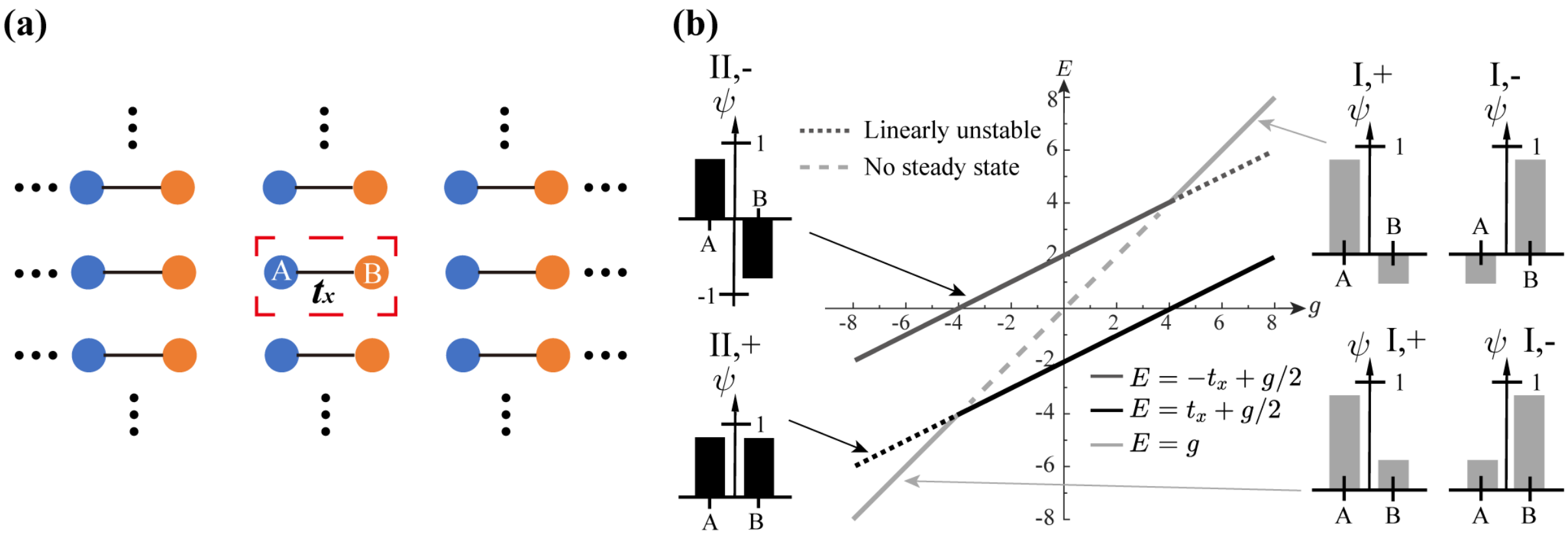

Figure S2. (a) Schematic of the stacked nonreciprocal SSH model in the molecular limit. A unit cell is circled by the red dashed line. (b) Two distinct types of nonlinear eigenstate spectra in the molecular limit (black and gray lines). The dashed line indicates that there are no type-I steady eigenstates within $|g| < -2t_x$ ($t_x = -2$ here). The dotted lines highlight the linearly unstable type-II eigenstates. The insets show the corresponding eigenstate profiles.

For type-II states, the normalized solutions are in the form of the following

$$\psi^{(\mathrm{II},\pm)} = \begin{pmatrix} \psi_A \\ \psi_B \end{pmatrix} = \frac{1}{\sqrt{2}}\begin{pmatrix} 1 \\ \pm 1 \end{pmatrix}, \qquad E = \pm t_x + \frac{g}{2}. \tag{S2.3}$$

The spectra are shown as black lines in Fig. S2(b), with the corresponding eigenstate profiles displayed on the left. According to linear stability analysis, solutions (II, +) [solutions (II, -)] are linearly unstable for $g < 2t_x$ ($g > -2t_x$) [4]. These unstable regimes are marked by the dotted black lines in Fig. S2(b). Note that g, E, and t_x share the same physical unit. For computational and presentation convenience, all parameters are treated as dimensionless throughout this work.

Section III. Numerical method for solving nonlinear equations

Here, we present details to solve Eq. (1) in the main text numerically. Specifically, we use self-consistent iteration, and here is the iterative process:

(i) Select an initial guess for the wave function $\psi^{(s=1)}$, where the superscript s denotes the iteration step. Successful initial guesses can be selected from the solutions that we introduced in Section II under the molecular limit.

(ii) Calculate the state-dependent nonlinear Hamiltonian $H_{\mathrm{NL}}(\psi^{(s)}) = H + g\left|\psi^{(s)}\right|^2$ including linear Hamiltonian H and nonlinearly-induced potential $g\left|\psi^{(s)}\right|^2$.

(iii) Solve the eigenstates ϕ_i of H_{NL}, where i is the state index.

(iv) Take the eigenstate $\phi_{i'}$ which closest to $\psi^{(s)}$ as the new state $\psi^{(s+1)}$ for the next iteration step. Here $\phi_{i'}$ satisfies $\left|\left|\langle\psi^{(s)}|\phi_{i'}\rangle\right| - 1\right| \leq \left|\left|\langle\psi^{(s)}|\phi_i\rangle\right| - 1\right|$ for all i.

(v) Repeat the processes of (ii), (iii), and (iv) until $\psi^{(s+1)}$ is close enough to $\psi^{(s)}$, i.e., $\left|\left|\langle\psi^{(s+1)}|\psi^{(s)}\rangle\right| - 1\right| < \epsilon_{nl}$ for an arbitrarily small quantity ϵ_{nl}. In the nonlinear iteration, we take $\epsilon_{nl} = 10^{-14}$.

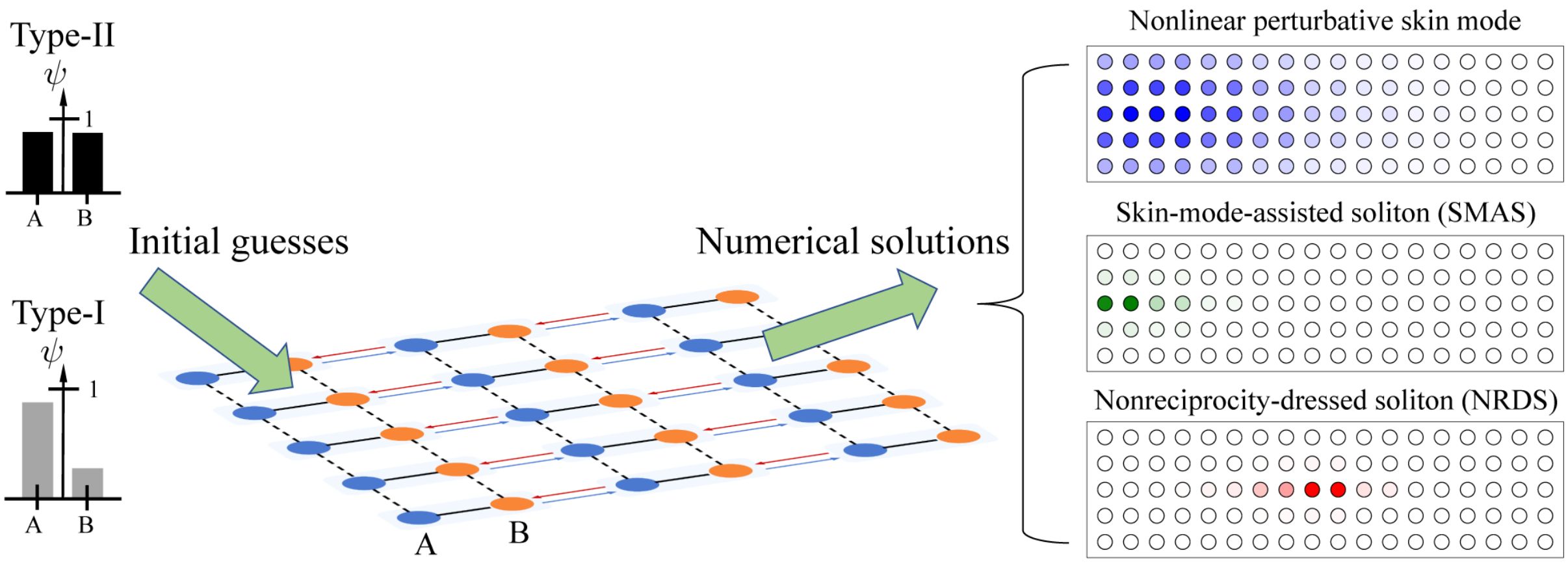

Figure S3. Schematic diagram showing the numerical process of solving nonlinear eigenstates in the stacked nonreciprocal SSH model under open boundary conditions.

The open boundary conditions are imposed by setting $\psi_{(N_x+1,n_y)} = \psi_{(0,n_y)} = \psi_{(n_x,N_y+1)} = \psi_{(n_x,0)} = 0$. We use initial guesses from different unit cells to perform nonlinear iterations across the entire lattice. Finally, we categorize the solutions into three families based on localization position, localization length, and mode profiles, as illustrated in Fig. S3.

Section IV. Regular perturbation theory

In this section, we demonstrate that nonlinear perturbative skin modes in the main text can be obtained by applying regular perturbation theory to their linear counterparts [5]. First, let us assume that ψ of a nonlinear perturbative skin mode in the following asymptotic form

$$\psi = \varepsilon\psi^{(0)} + \varepsilon^3\psi^{(1)} + \mathcal{O}(\varepsilon^5), \tag{S4.1}$$

where $\psi^{(0)}$ is the linear eigenstate (right eigenvector) of the unperturbed system. Due to the gauge freedom that ψ and $\tilde{\psi} = e^{i\theta}\psi$ with an arbitrary phase θ correspond to the same eigenvalue, E, for ψ with the form of Eq. (S4.1), must have the following asymptotic form

$$E = E^{(0)} + \varepsilon^2 E^{(1)} + \mathcal{O}(\varepsilon^4), \tag{S4.2}$$

where ε can be determined from $\sum_{\boldsymbol{n}}|\psi_{\boldsymbol{n}}|^2 = 1$. Substitute Eqs. (S4.1) and (S4.2) into the following nonlinear equation

$$E\psi = H\psi + g|\psi|^2\psi, \tag{S4.3}$$

we obtain

$$\left(E^{(0)} + \varepsilon^2 E^{(1)}\right)\left(\varepsilon\psi^{(0)} + \varepsilon^3\psi^{(1)}\right) = H\left(\varepsilon\psi^{(0)} + \varepsilon^3\psi^{(1)}\right) + g\varepsilon^3\left|\psi^{(0)}\right|^2\psi^{(0)}. \tag{S4.4}$$

Assuming that

$$\begin{aligned} E^{(0)}\psi^{(0)} &= H\psi^{(0)}, \\ E^{(0)}\psi^{(1)} &= H\psi^{(1)}, \end{aligned} \tag{S4.5}$$

we further obtain

$$E^{(1)}\psi^{(0)} = g\left|\psi^{(0)}\right|^2\psi^{(0)}. \tag{S4.6}$$

We then multiply both sides of Eq. (S4.6) by the left eigenvector $\langle\psi^{(0)}|$ to obtain the first-order energy correction

$$E^{(1)} = g\left\langle\psi^{(0)}\middle|\left|\psi^{(0)}\right|^2\psi^{(0)}\right\rangle. \tag{S4.7}$$

The above results show that if we gradually increase the nonlinearity from $g = 0$, a family of nonlinear modes can emerge from their corresponding linear modes, and their energies are modified by Eq. (S4.2) starting from $E^{(0)}$ of the linear mode. As shown in the left panel of Fig. S4(a), the representative spectra of nonlinear perturbative skin modes (blue circles) are in good

agreement with those (green line) obtained by Eq. (S4.2), which demonstrates that nonlinear perturbative skin modes can be treated as emerging from the skin modes under open boundaries. We also examine the case with a larger t_y, as displayed in Fig. S4(b), the perturbation theory works as well.

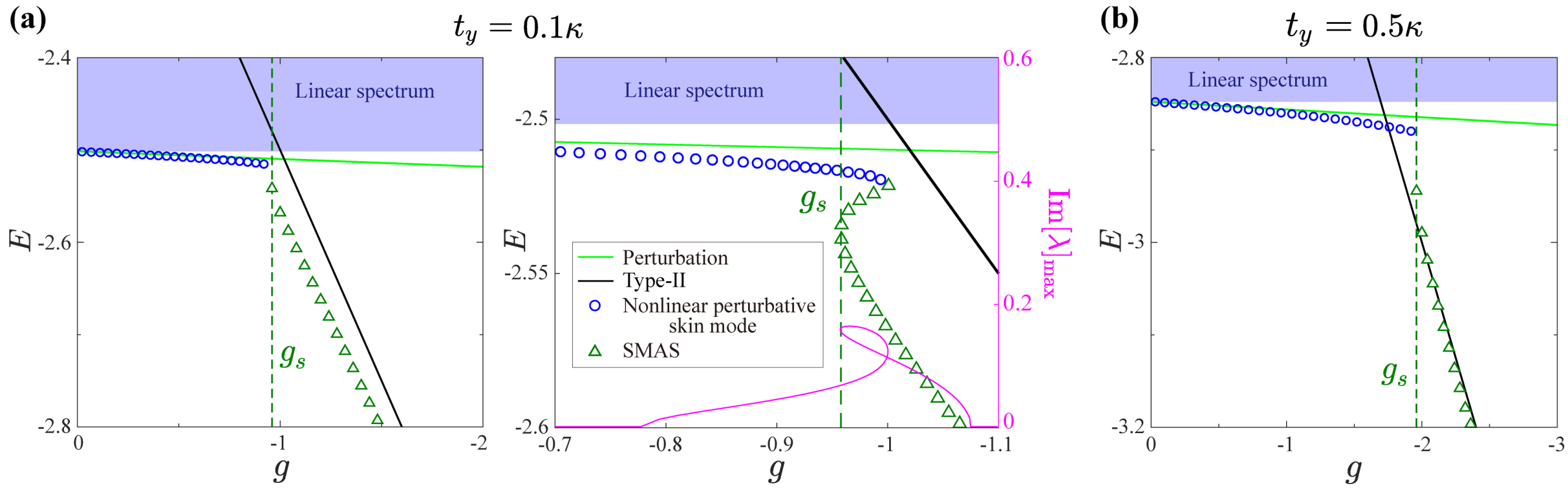

Figure S4. (a,b) Spectra of nonlinear perturbative skin modes (circles) and SMASs (triangles) for $\gamma = 0.5$, with $t_y = 0.1\kappa$ (a) and $t_y = 0.5\kappa$ (b). The black line indicates the analytical spectra for Type-II states in the molecular limit. The solid green line is obtained from regular perturbation theory, with $E^{(0)}$ taken as the lowest energy of the linear spectra under open boundary conditions. The left panel of (a) is reproduced from Fig. 2(c) in the main text, while the right panel displays a magnified view near g_s. The magenta line exhibits $\text{Im}[\lambda]_{\text{max}}$, the maximum imaginary part of the eigenvalues of the stability matrix for the considered modes.

The right panel of Fig. S4(a) displays a magnified view of the spectra and linear stability analysis near g_s. For $g < g_s$, nonlinear perturbative skin modes can have steady-state solutions within a small portion of the parameter range, but these solutions are linearly unstable, as indicated by $\text{Im}[\lambda]_{\text{max}} \neq 0$. When $|g|$ further increases, no steady-state solutions exist for this solution family. Therefore, for $g < g_s$, steady-state solutions for nonlinear perturbative skin modes exist only in a narrow parameter range and are linearly unstable. Within this parameter range, skin-mode-assisted solitons (SMASs) emerge as steady-state solitons instead.

Figure S5 displays the mode profile of nonlinear eigenstates $|\psi_n|$ for $\gamma = 0.5$ and $\gamma = 0$ in the weak nonlinear region. The upper panel depicts a nonlinear perturbative skin mode at $\gamma = 0.5$, which remains localized near the left boundary, closely resembling its linear counterpart in Fig. S1(d). In contrast, the lower panel shows the nonlinear eigenstate when $\gamma = 0$, which remains delocalized. Both are similar to their linear counterparts, validating the perturbation theory.

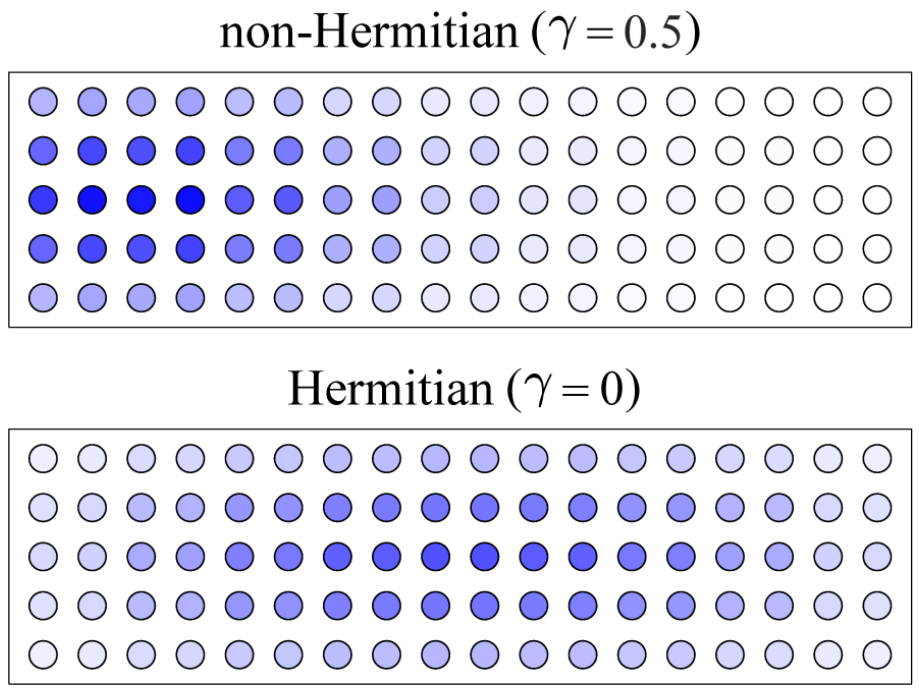

Figure S5. $|\psi_{\boldsymbol{n}}|$ for a typical nonlinear perturbative skin mode at $\gamma = 0.5$ (upper panel) and a delocalized mode at $\gamma = 0$ (lower panel) for $g = -0.6$. The color shading represents the amplitude $|\psi_{\boldsymbol{n}}|$.

Section V. From type-II to type-I solitons

In this section, we detail the transformation from type-II to type-I solitons. If we treat them as equiamplitude and non-equiamplitude solitons, then the transformation has a clear definition, and no coexistence of type-I and type-II solitons exists.

From the soliton profile, type-II solitons correspond to those with equiamplitudes ($|\psi_A| = |\psi_B|$), while type-I solitons imply the non-equiamplitude soliton ($|\psi_A| \neq |\psi_B|$). In this sense, type-I and type-II solitons should be understood as two subclasses of each soliton family, rather than distinct families. We distinguish these subclasses using a specific value in the g-parameter, denoted as g_t.

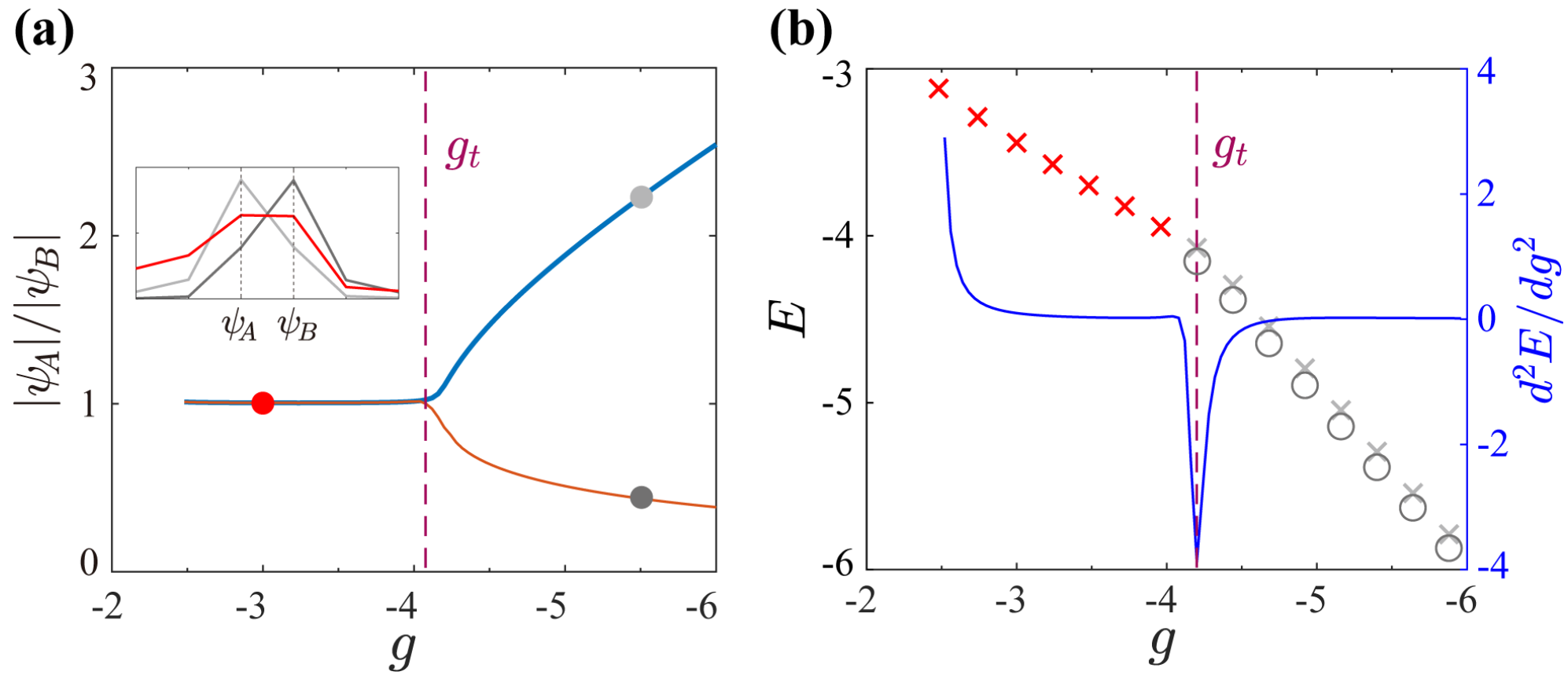

Figure S6. (a) The ratio $|\psi_A|/|\psi_B|$ of NRDSs at two main sites, as a function of g (cyan and orange lines). The solid lines in the inset are the soliton profiles under three g-parameters (corresponding to the three dots), and the black dashed lines mark the two main sites we chose. (b) Spectra of type-II NRDSs (red cross markers) and the two branches of type-I NRDSs (gray cross markers and circles). The blue line displays d^2E/d^2g, whose peak indicates the inflection point of the spectra. The dashed amaranth line marks g_t.

To distinguish between the two types of solitons, we introduce an indicator $|\psi_A|/|\psi_B|$, using nonreciprocity-dressed solitons (NRDSs) as an example. The solid cyan and orange lines in Fig. S6(a) display the computed $|\psi_A|/|\psi_B|$ for the linearly stable and steady-state NRDSs found in the lattice. For $|g| < |g_t|$, the NRDS exhibits equiamplitude, i.e., $\frac{|\psi_A|}{|\psi_B|} = 1$, and thus belongs to type-II. When $|g| > |g_t|$, the NRDS bifurcates into two branches with $\frac{|\psi_A|}{|\psi_B|} \neq 1$. The inset shows the corresponding soliton profile, indicating that the equiamplitude soliton evolves to two non-equiamplitude solitons, which respectively concentrate at the sites A and B. Consequently, the NRDS branches for $|g| > |g_t|$ belong to type-I.

To further pin down g_t, we display the spectra for both types of NRDSs, as shown by cross markers and circles in Fig. S6(b). The slope obviously experiences a dramatic change when g varies, and hence, we calculate the second derivative of E with respect to g, as shown by the blue line in Fig. S6(b). An apparent peak exists, and we refer to its g value as g_t [dashed lines in Figs. S6(a) and S6(b)]. This g_t separates two subclasses of NRDSs if we follow the equiamplitude criterion. For the lattice under consideration, the value of g_t is close to the crossover point of two types of eigenstates in the molecular limit [$g = -4$ in Fig. S2(b)].

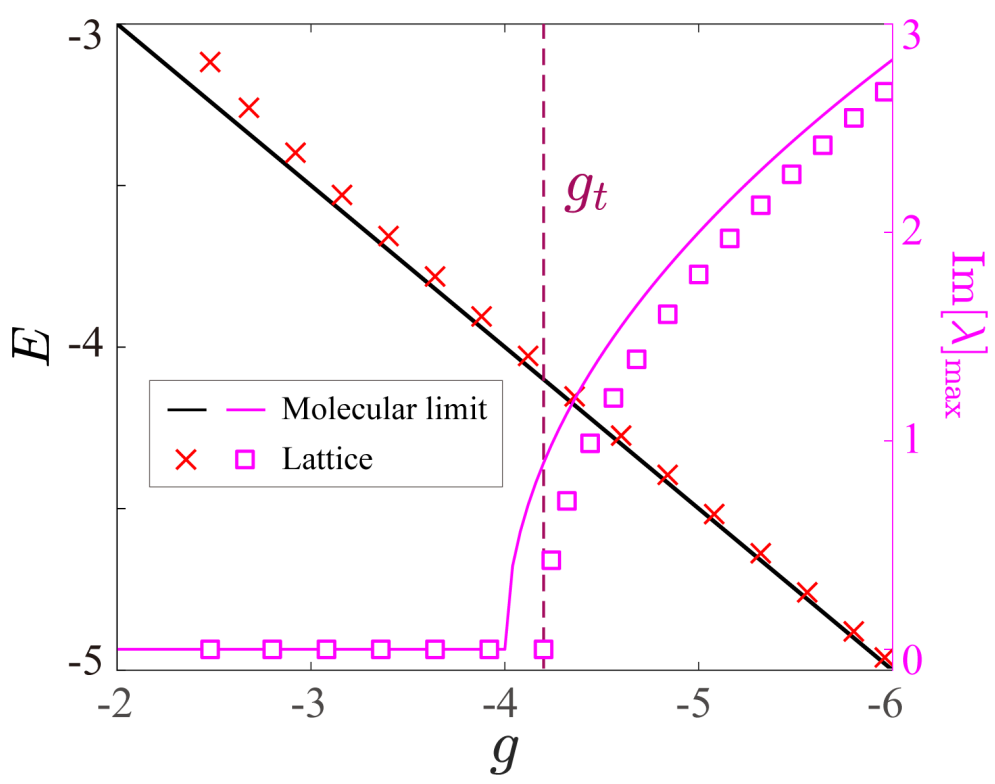

Figure S7. Spectra and $\mathrm{Im}[\lambda]_{\max}$ of type-II NRDSs (red crosses and open squares) in a real lattice. The dashed line marks g_t. The black and magenta lines indicate analytical spectra and $\mathrm{Im}[\lambda]_{\max}$ of the type-II states in the molecular limit.

Thus, type-II solitons are the first stable, steady-state solutions to appear in the lattice. Once the nonlinearity strength g exceeds the value g_t, they evolve to type-I solitons. To further verify this, we numerically compute type-II solitons for $g < g_t$. As shown in Fig. S7, analogous to the linear instability of type-II states in the molecular limit for $g < 2t_x$ (solid black line, instability indicated by magenta line), type-II solitons (red crosses) exist for $g < g_t$ (where $g_t \cong 2t_x$) but are linearly unstable (open squares). Therefore, Fig. 2(c) in the main text

does not include type-II solitons when $g < g_t$ because they do not obey the linearly stable and steady-state condition.

Section VI. Linear stability analysis

To perform the linear stability analysis [6], we write down the time-dependent equation governed by Eq. (1) in the main text

$$i\frac{d\Psi_{\boldsymbol{n}}}{dt} = H_{\boldsymbol{nm}}\Psi_{\boldsymbol{m}} + g|\Psi_{\boldsymbol{n}}|^2\Psi_{\boldsymbol{n}}, \tag{S6.1}$$

where $\Psi_{\boldsymbol{n}}(t)$ is in the time domain. Equation (S6.1) recovers Eq. (1) when $\Psi_{\boldsymbol{n}}(t) \to e^{-iEt}\psi_{\boldsymbol{n}}$. We then suppose perturbations to a given eigenstate $\psi_{\boldsymbol{n}}$ as $\Psi_{\boldsymbol{n}}(t) = [\psi_{\boldsymbol{n}} + \epsilon v_{\boldsymbol{n}}(t)]e^{-iEt}$, where $\epsilon \ll 1$. By substituting it into Eq. (S6.1) and assuming $v_{\boldsymbol{n}}(t) = p_{\boldsymbol{n}}e^{-i\lambda t} + q_{\boldsymbol{n}}^* e^{i\lambda^* t}$, we ultimately obtain the linear stability eigenvalue problem

$$Z\begin{pmatrix}\boldsymbol{p}\\ \boldsymbol{q}\end{pmatrix} = \lambda\begin{pmatrix}\boldsymbol{p}\\ \boldsymbol{q}\end{pmatrix}, \tag{S6.2}$$

where Z is the stability matrix with λ being its eigenvalue. The vectors $\boldsymbol{p}$ and $\boldsymbol{q}$ collect the amplitudes $p_{\boldsymbol{n}}$ and $q_{\boldsymbol{n}}$ over all lattice sites $\boldsymbol{n}$.

When all eigenvalues λ of Z are real, the corresponding mode is linearly stable; otherwise, it is unstable. We thus use the maximum imaginary part of λ, denoted by $\mathrm{Im}[\lambda]_{\max}$, as a stability indicator. The magenta line in Fig. 2(c) in the main text and the color map in Fig. S8(a) show $\mathrm{Im}[\lambda]_{\max}$ for the nonlinear perturbative skin modes, SMASs, and NRDSs presented therein. Evidently, instability occurs only near the contact between nonlinear perturbative skin modes and SMASs, indicating a stability gap around g_s.

To verify the results of stability analysis, we investigate system dynamics by using the eigenstate with perturbations as the initial excitation, i.e., $\Psi_{\boldsymbol{n}}(t=0) = \psi_{\boldsymbol{n}} + \epsilon v_{\boldsymbol{n}}(t=0)$ with $v_{\boldsymbol{n}}$ being the eigenvector corresponding to $\mathrm{Im}[\lambda]_{\max}$. Figures S8(b) to S8(d) display the evolution of one linear skin mode and two nonlinear perturbative skin modes, while Figs. S8(e) to S8(g) depict those for one SMAS and two NRDSs. Clearly, the transformation from nonlinear perturbative skin modes to SMASs undergoes a stability reentrance [Figs. S8(c) to S8(e)]. We also demonstrate, through dynamical simulation, that all the considered NRDSs are linearly stable [Figs. S8(f) and S8(g)].

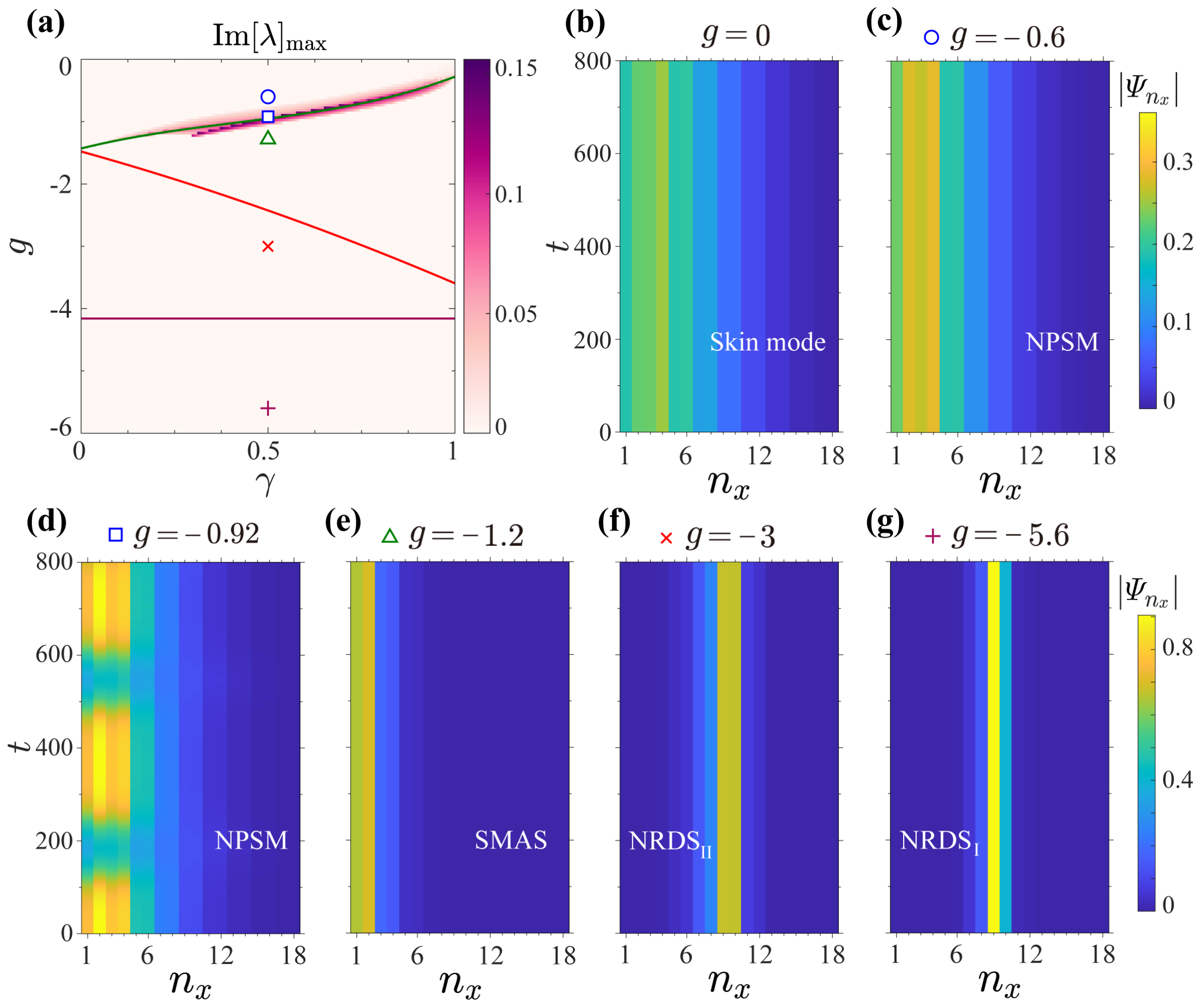

Figure S8. (a) Stability diagram extending over a broader parameter range than the threshold diagram of Fig. 3(a) in the main text. The color map shows $\mathrm{Im}[\lambda]_{\max}$ for the modes under consideration, while the green, red, and amaranth lines display g_s, g_n, and g_t, respectively. (b)-(g) Time evolution of $|\Psi_{(n_x,n_y=3)}(t)|$ for various states: (b) stable skin mode; (c) stable nonlinear perturbative skin mode (NPSM); (d) unstable NPSM; (e) stable SMAS; (f) stable type-II NRDS ($\mathrm{NRDS_{II}}$); (g) stable type-I NRDS ($\mathrm{NRDS_{I}}$). (b)-(d) share the upper color bar, while (e)-(g) share the lower one. Their corresponding g values are labelled herein, $\epsilon = 10^{-3}$, and t is in units of $1/2|\kappa|$. The simulations are performed with open boundary conditions using the Runge-Kutta method.

Section VII. Wannier-projected effective nonlinear Hamiltonian

In this section, we present details for constructing the Wannier-projected effective nonlinear Hamiltonian (Sec. VII A). Section VII B discusses how to solve the soliton thresholds g_s and g_n by retaining two dominant Wannier functions to truncate the nonlinear Hamiltonian.

A. Details of the nonlinear Hamiltonian

First, we expand the real-space $\psi_{\boldsymbol{n}}$ in a Wannier basis [7–9]

$$\psi_{\boldsymbol{n}} = \sum_{\boldsymbol{R},\alpha} c_{\boldsymbol{R},\alpha} w^{\mathrm{R}}_{\boldsymbol{R},\alpha,\boldsymbol{n}}, \tag{S7.1}$$

where $c_{\boldsymbol{R},\alpha}$ are expansion coefficients, $\boldsymbol{R} = (R_x, R_y)$ denotes the real-space lattice vector, α is the band index, and $w^{\mathrm{R}}_{\boldsymbol{R},\alpha,\boldsymbol{n}}$ are the Wannier functions constructed from right eigenvectors (with superscript R; L for left). For the non-Hermitian Hamiltonian H, the right (left) eigenvectors $|\psi^{\mathrm{R}}\rangle$ ($\langle\psi^{\mathrm{L}}|$) are defined as $H|\psi^{\mathrm{R}}\rangle = E|\psi^{\mathrm{R}}\rangle$ ($\langle\psi^{\mathrm{L}}|H = E\langle\psi^{\mathrm{L}}|$). Unlike the Hermitian systems with $|\psi^{\mathrm{R}}\rangle^{\dagger} = \langle\psi^{\mathrm{L}}|$, for non-Hermitian systems, $|\psi^{\mathrm{R}}\rangle^{\dagger} \neq \langle\psi^{\mathrm{L}}|$ and satisfies the following biorthogonal normalization

$$\langle\psi^{\mathrm{L}}_{\alpha'}|\psi^{\mathrm{R}}_{\alpha}\rangle = \delta_{\alpha',\alpha}. \tag{S7.2}$$

Wannier functions form Fourier pairs with Bloch functions $u_{\boldsymbol{k},\alpha,\boldsymbol{n}}$ ($\boldsymbol{k}$ are the wave vectors in momentum space). For non-Hermitian SSH lattices under consideration here, the Wannier functions constructed from left and right eigenstates are

$$w^{\mathrm{L}}_{\boldsymbol{R},\alpha,\boldsymbol{n}} = \frac{1}{\sqrt{N}}\sum_{\boldsymbol{k}} e^{-i\boldsymbol{k}\cdot\boldsymbol{R}} u^{\mathrm{L}}_{\boldsymbol{k},\alpha,\boldsymbol{n}},$$
$$u^{\mathrm{L}}_{\boldsymbol{k},\alpha,\boldsymbol{n}} = \frac{1}{\sqrt{N}}\sum_{\boldsymbol{R}} e^{i\boldsymbol{k}\cdot\boldsymbol{R}} w^{\mathrm{L}}_{\boldsymbol{R},\alpha,\boldsymbol{n}}, \tag{S7.3}$$
$$w^{\mathrm{R}}_{\boldsymbol{R},\alpha,\boldsymbol{n}} = \frac{1}{\sqrt{N}}\sum_{\boldsymbol{k}} e^{-i\boldsymbol{k}\cdot\boldsymbol{R}} u^{\mathrm{R}}_{\boldsymbol{k},\alpha,\boldsymbol{n}},$$
$$u^{\mathrm{R}}_{\boldsymbol{k},\alpha,\boldsymbol{n}} = \frac{1}{\sqrt{N}}\sum_{\boldsymbol{R}} e^{i\boldsymbol{k}\cdot\boldsymbol{R}} w^{\mathrm{R}}_{\boldsymbol{R},\alpha,\boldsymbol{n}}, \tag{S7.4}$$

where N is the total number of unit cells. Clearly, they satisfy the following biorthogonal normalization

$$\sum_{\boldsymbol{n}} w^{\mathrm{L}}_{\boldsymbol{R}',\alpha',\boldsymbol{n}} w^{\mathrm{R}}_{\boldsymbol{R},\alpha,\boldsymbol{n}} = \delta_{\boldsymbol{R}',\boldsymbol{R}}\delta_{\alpha',\alpha}. \tag{S7.5}$$

Figures S9(a) and S9(b) display the real-space distribution of maximally localized Wannier functions of the valence band ($\alpha = 1$) for Hermitian ($\gamma = 0$) and non-Hermitian ($\gamma = 0.5$) lattices, respectively. At $\gamma = 0.5$, the Wannier functions exhibit asymmetry but nonetheless remain largely confined to a single unit cell, similar to the profiles of the solitons.

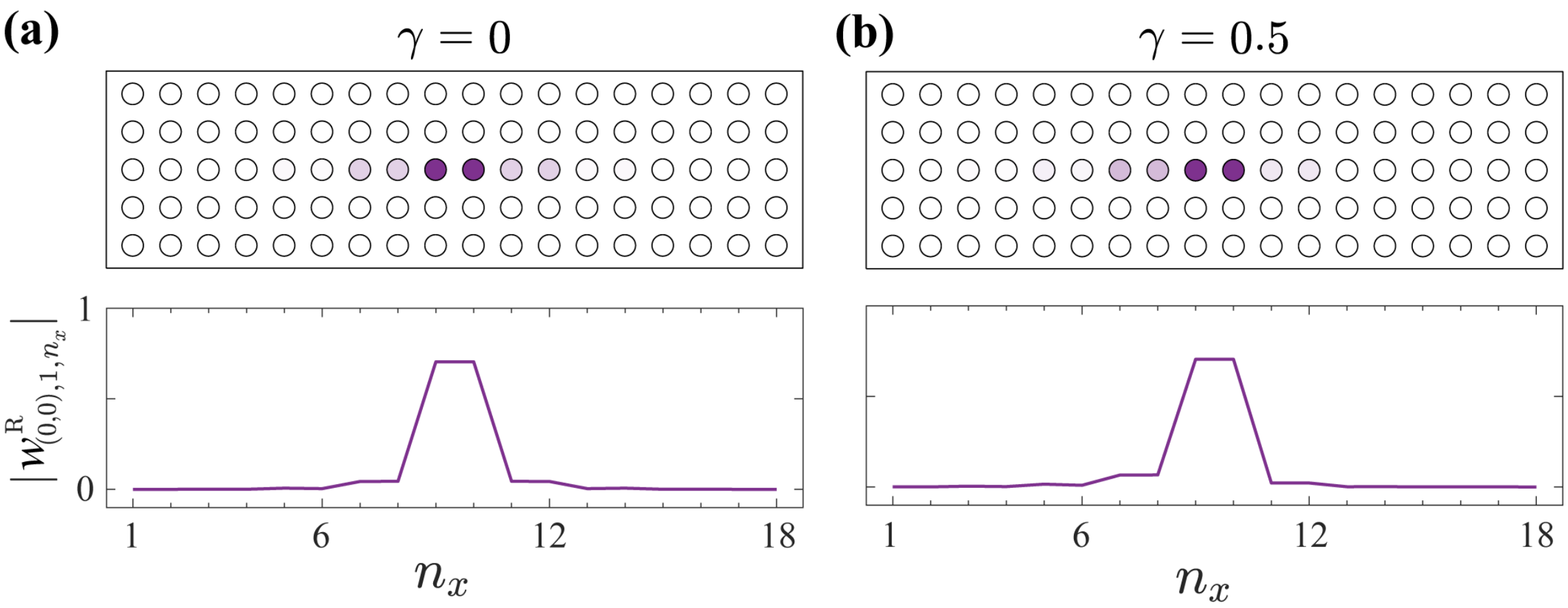

Figure S9. (a,b) Spatial distribution of $|w^{\mathrm{R}}_{(0,0),1,\boldsymbol{n}}|$ (upper panel) for $\gamma = 0$ (a) and $\gamma = 0.5$ (b). The solid lines in the lower panels display the corresponding $|w^{\mathrm{R}}_{(0,0),1,(n_x,n_y=3)}|$. Here, we set the lattice vector of the unit cell located in the center of the lattice as $\boldsymbol{R} = (0,0)$.

To get the Wannier-projected effective nonlinear Hamiltonian, we substitute Eq. (S7.1) into the real-space discrete nonlinear Schrödinger equation

$$E\psi_{\boldsymbol{n}} = \sum_{\boldsymbol{m}} H_{\boldsymbol{mn}}\psi_{\boldsymbol{m}} + g|\psi_{\boldsymbol{n}}|^2\psi_{\boldsymbol{n}}, \tag{S7.6}$$

and multiply both sides of Eq. (S7.6) by $w^{\mathrm{L}}_{\boldsymbol{R}',\alpha',\boldsymbol{n}}$. Next, by summing all the sites $\boldsymbol{n}$ and using the biorthogonal relation [Eq. (S7.5)], we obtain

$$\begin{aligned} Ec_{\boldsymbol{R}',\alpha'} &= \sum_{\boldsymbol{R},\alpha} c_{\boldsymbol{R},\alpha} \sum_{\boldsymbol{n},\boldsymbol{m}} w^{\mathrm{L}}_{\boldsymbol{R}',\alpha',\boldsymbol{n}} H_{\boldsymbol{nm}} w^{\mathrm{R}}_{\boldsymbol{R},\alpha,\boldsymbol{m}} \\ &+ \sum_{\substack{\boldsymbol{R},\boldsymbol{R}'',\boldsymbol{R}''' \\ \alpha,\alpha'',\alpha'''}} gW^{\alpha,\alpha',\alpha'',\alpha'''}_{\boldsymbol{R},\boldsymbol{R}',\boldsymbol{R}'',\boldsymbol{R}'''} c^*_{\boldsymbol{R},\alpha} c_{\boldsymbol{R}'',\alpha''} c_{\boldsymbol{R}''',\alpha'''}, \end{aligned} \tag{S7.7}$$

where $W^{\alpha,\alpha',\alpha'',\alpha'''}_{\boldsymbol{R},\boldsymbol{R}',\boldsymbol{R}'',\boldsymbol{R}'''}$ is the overlap integral of four Wannier functions, i.e.,

$$W^{\alpha,\alpha',\alpha'',\alpha'''}_{\boldsymbol{R},\boldsymbol{R}',\boldsymbol{R}'',\boldsymbol{R}'''} = \sum_{\boldsymbol{n}} \left(w^{\mathrm{R}}_{\boldsymbol{R},\alpha,\boldsymbol{n}}\right)^* w^{\mathrm{L}}_{\boldsymbol{R}',\alpha',\boldsymbol{n}} w^{\mathrm{R}}_{\boldsymbol{R}'',\alpha'',\boldsymbol{n}} w^{\mathrm{R}}_{\boldsymbol{R}''',\alpha''',\boldsymbol{n}}. \tag{S7.8}$$

To simplify the first term in Eq. (S7.7), we consider the following eigenvalue equation

$$E_{\boldsymbol{k},\alpha} u_{\boldsymbol{k},\alpha,\boldsymbol{n}} = \sum_{\boldsymbol{m}} H_{\boldsymbol{nm}} u_{\boldsymbol{k},\alpha,\boldsymbol{n}}, \tag{S7.9}$$

and expand $E_{\boldsymbol{k},\alpha}$ as the following Fourier series

$$E_{\boldsymbol{k},\alpha} = \sum_{\tilde{\boldsymbol{R}}} e^{i\boldsymbol{k}\cdot\tilde{\boldsymbol{R}}} \epsilon_{\tilde{\boldsymbol{R}},\alpha}, \tag{S7.10}$$

where $\epsilon_{\tilde{\boldsymbol{R}},\alpha}$ is the Fourier coefficient of $E_{\boldsymbol{k},\alpha}$, i.e., $\epsilon_{\tilde{\boldsymbol{R}},\alpha} = \left[\sum_{\boldsymbol{k}} e^{-i\boldsymbol{k}\cdot\tilde{\boldsymbol{R}}} E_{\boldsymbol{k},\alpha}\right]/N$. Using Eqs. (S7.4), (S7.5), (S7.9), and (S7.10), we can simplify the first term of Eq. (S7.7) as

$$\sum_{\boldsymbol{R},\alpha} c_{\boldsymbol{R},\alpha} \sum_{\boldsymbol{n},\boldsymbol{m}} w^{\mathrm{L}}_{\boldsymbol{R}',\alpha',\boldsymbol{n}} H_{\boldsymbol{nm}} w^{\mathrm{R}}_{\boldsymbol{R},\alpha,\boldsymbol{m}} = \sum_{\boldsymbol{R}} \epsilon_{\boldsymbol{R}-\boldsymbol{R}',\alpha'} c_{\boldsymbol{R},\alpha'} \,. \tag{S7.11}$$

Substituting Eq. (S7.11) into Eq. (S7.7), we obtain

$$E c_{\boldsymbol{R},\alpha} = \sum_{\boldsymbol{R}'} \epsilon_{\boldsymbol{R}'-\boldsymbol{R},\alpha} c_{\boldsymbol{R}',\alpha} + \sum_{\substack{\boldsymbol{R}',\boldsymbol{R}'',\boldsymbol{R}''' \\ \alpha',\alpha'',\alpha'''}} g W^{\alpha,\alpha',\alpha'',\alpha'''}_{\boldsymbol{R}',\boldsymbol{R},\boldsymbol{R}'',\boldsymbol{R}'''} c^{*}_{\boldsymbol{R}',\alpha'} c_{\boldsymbol{R}'',\alpha''} c_{\boldsymbol{R}''',\alpha'''}. \tag{S7.12}$$

This is not a matrix form, and we now make the following reasonable assumptions:

1. Ignore the nonlinear band-to-band coupling terms in Eq. (S7.12) and consider only a single band α. This is because the energy bands of our system are nondegenerate at any momentum $\boldsymbol{k}$, and the maximum nonlinear contribution $\max[g|\psi_{\boldsymbol{n}}|^2]$ to Hamiltonian within the g of interest is less than the linear band gaps.
2. Keep diagonal terms in $W^{\alpha,\alpha',\alpha'',\alpha'''}_{\boldsymbol{R}',\boldsymbol{R},\boldsymbol{R}'',\boldsymbol{R}'''}$, namely $\boldsymbol{R} = \boldsymbol{R}' = \boldsymbol{R}'' = \boldsymbol{R}'''$. Because our bands are not topological, Wannier functions are not only exponentially attenuated at infinity but also decay immediately away from the Wannier center.

Applying the above two assumptions, Eq. (S7.12) takes the following completely simplified form

$$E c_{\boldsymbol{R},\alpha} = \sum_{\boldsymbol{R}'} \epsilon_{\boldsymbol{R}'-\boldsymbol{R},\alpha} c_{\boldsymbol{R}',\alpha} + g W^{\alpha,\alpha,\alpha,\alpha}_{\boldsymbol{R},\boldsymbol{R},\boldsymbol{R},\boldsymbol{R}} \left|c_{\boldsymbol{R},\alpha}\right|^2 c_{\boldsymbol{R},\alpha} \,. \tag{S7.13}$$

Since Eq. (S7.10) is under periodic boundary conditions, Eq. (S7.13) is now only valid for the periodic-boundary clusters. However, we can apply open boundary conditions by vanishing boundary hoppings and then formally rewrite Eq. (S7.13) as

$$E c_{\boldsymbol{R},\alpha} = \sum_{\boldsymbol{R}'} \epsilon_{\boldsymbol{R},\boldsymbol{R}',\alpha} c_{\boldsymbol{R}',\alpha} + g W^{\alpha,\alpha,\alpha,\alpha}_{\boldsymbol{R},\boldsymbol{R},\boldsymbol{R},\boldsymbol{R}} \left|c_{\boldsymbol{R},\alpha}\right|^2 c_{\boldsymbol{R},\alpha} \,. \tag{S7.14}$$

It can be recast into the standard form of the Wannier-projected effective nonlinear Hamiltonian, Eq. (3) in the main text. The open boundary conditions are implemented numerically by setting the boundary elements in $\epsilon_{\boldsymbol{R},\boldsymbol{R}'}$ to zero, i.e., if $\boldsymbol{R}'$ is far away from $\boldsymbol{R}$, we set $\epsilon_{\boldsymbol{R},\boldsymbol{R}'} = 0$; otherwise, $\epsilon_{\boldsymbol{R},\boldsymbol{R}'} = \epsilon_{\boldsymbol{R}'-\boldsymbol{R}} = \left[\sum_{\boldsymbol{k}} e^{-i\boldsymbol{k}\cdot(\boldsymbol{R}'-\boldsymbol{R})} E_{\boldsymbol{k}}\right]/N$.

We supplementally examine a nonlinear perturbative skin mode and an SMAS localized in a different unit cell using the nonlinear Hamiltonian introduced above. Unlike the leftmost SMAS presented in the main text, the SMAS studied here resides deeper in the bulk. The mode profiles are shown by open circles in Figs. S10(a) and S10(b). Figures S10(c) and S10(d) depict

expansion coefficients $c_{\boldsymbol{R}}$ throughout the entire lattice corresponding to Figs. S10(a) and S10(b). The solid lines show the results obtained by numerically solving the real-space nonlinear eigenvalue equation [Eq. (1) in the main text]. The excellent agreement between the open circles and solid lines further validates the Wannier-projected effective nonlinear Hamiltonian.

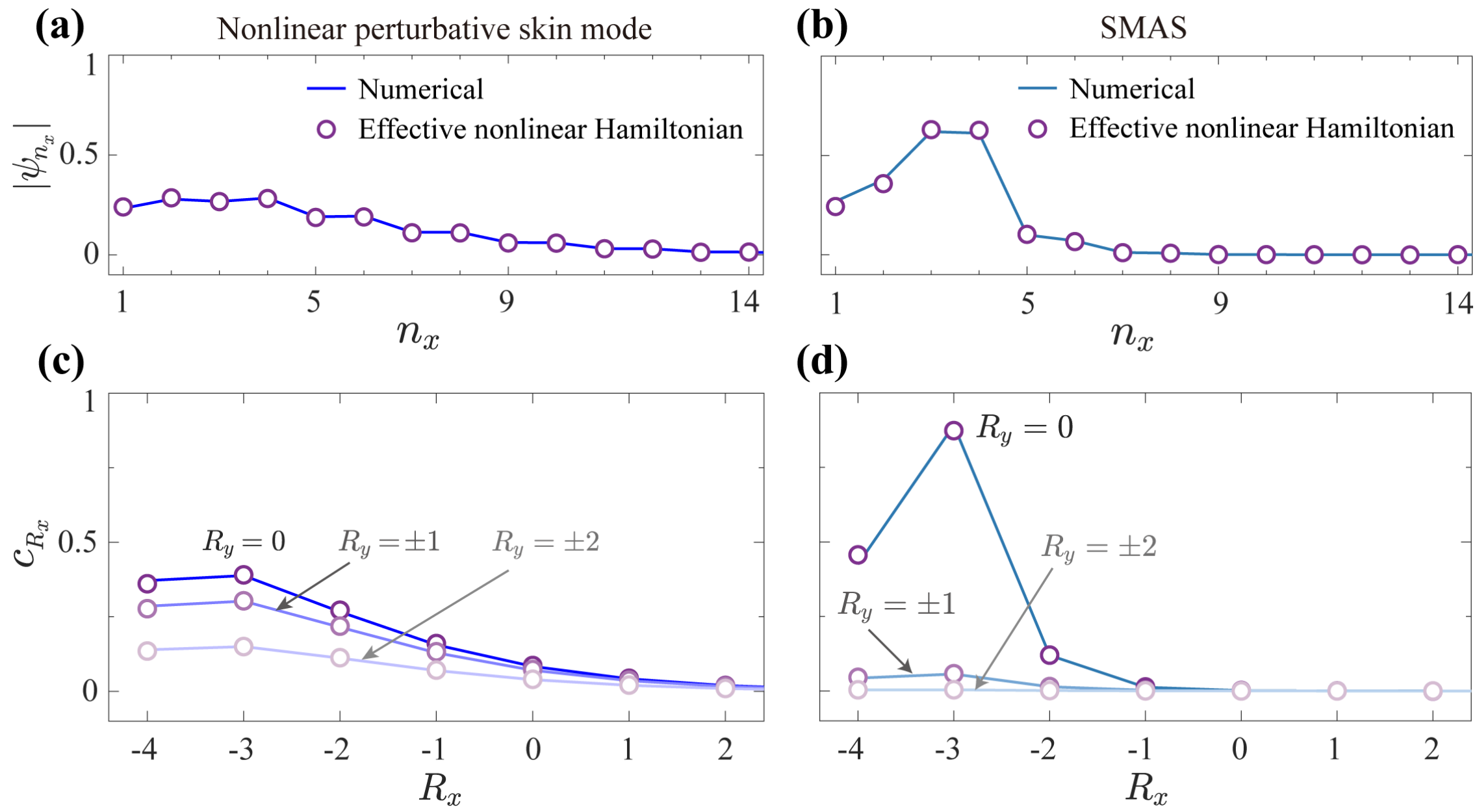

Figure S10. (a-d) Comparison of $|\psi_{(n_x,n_y=3)}|$ (a,b) and $c_{(R_x,R_y=0)}$ (c,d) for a nonlinear perturbative skin mode at $g = -0.6$ (a,c) and an SMAS at $g = -2.2$ (b,d). Solid lines in (a,b) [(c,d)] are obtained by numerically solving the real-space nonlinear eigenvalue equation (obtained by numerical projection onto the Wannier basis). Open circles are all calculated from the nonlinear Hamiltonian.

B. Analytical forms of soliton thresholds

Figure 3(b) in the main text shows that both SMASs and NRDSs can be reconstructed by using several Wannier functions. Therefore, we retain the two leading Wannier functions and aim to work out g_s and g_n analytically. For g_s, considering the expansion coefficients $c_{\boldsymbol{R}}$ being nonzero at $c_{(-4,0)}$ and $c_{(-3,0)}$, Eq. (S7.14) can be approximated as

$$Ec_{(-4,0)} = gWc_{(-4,0)}^3 + \epsilon_{(-4,0),(-4,0)}c_{(-4,0)} + \epsilon_{(-4,0),(-3,0)}c_{(-3,0)}, \tag{S7.15}$$

$$Ec_{(-3,0)} = gWc_{(-3,0)}^3 + \epsilon_{(-3,0),(-3,0)}c_{(-3,0)} + \epsilon_{(-3,0),(-4,0)}c_{(-4,0)}, \tag{S7.16}$$

where $W = W_{\boldsymbol{R},\boldsymbol{R},\boldsymbol{R},\boldsymbol{R}}^{\alpha,\alpha,\alpha,\alpha}$. Substituting $c_{(-3,0)} = \varsigma c_{(-4,0)}$ into Eqs. (S7.15) and (S7.16), we obtain

$$\left(E - \epsilon^{(0)} - \varsigma\epsilon_{\leftarrow}\right)c_{(-4,0)} = \eta c_{(-4,0)}^3, \tag{S7.17}$$

$$\left(E - \epsilon^{(0)}\right)c_{(-3,0)} = \eta c_{(-3,0)}^3 + \epsilon_{\rightarrow}c_{(-4,0)}, \tag{S7.18}$$

where $\eta = gW$, $\epsilon_{(-4,0),(-4,0)} = \epsilon_{(-3,0),(-3,0)} = \epsilon^{(0)}$, $\epsilon_{(-4,0),(-3,0)} = \epsilon_{\leftarrow}$, and $\epsilon_{(-3,0),(-4,0)} = \epsilon_{\rightarrow}$. From Eq. (S7.17), we obtain $c_{(-4,0)}^2 = (E' - \varsigma\epsilon_{\leftarrow})/\eta$, where $E' = E - \epsilon^{(0)}$.

Consider the normalization

$$c_{(-3,0)}^2 = 1 - c_{(-4,0)}^2 = 1 - \frac{1}{\eta}(E' - \varsigma\epsilon_{\leftarrow}). \tag{S7.19}$$

We reexpress Eq. (S7.18) as

$$\left(E' - \eta c_{(-3,0)}^2\right)^2 c_{(-3,0)}^2 = \epsilon_{\rightarrow}{}^2 c_{(-4,0)}^2. \tag{S7.20}$$

Substituting $c_{(-4,0)}^2$ and $c_{(-3,0)}^2$ into Eq. (S7.20) arrives

$$\begin{gathered} 4E''^3 + 4(\varsigma\epsilon_{\leftarrow} - 2\eta)E''^2 \\ +[(\varsigma\epsilon_{\leftarrow} - \eta)^2 - 4\eta(\varsigma\epsilon_{\leftarrow} - \eta) + \epsilon_{\rightarrow}{}^2]E'' - (\varsigma\epsilon_{\leftarrow} - \eta)^2\eta = 0, \end{gathered} \tag{S7.21}$$

where $E'' = E' - \varsigma\epsilon_{\leftarrow}$. Equation (S7.21) is a cubic equation about E'', and its discriminant Δ has the following form

$$\begin{gathered} \Delta = 16\epsilon_{\rightarrow}{}^2\eta^4 + 32\epsilon_{\rightarrow}{}^2\varsigma\epsilon_{\leftarrow}\eta^3 - 176\epsilon_{\rightarrow}{}^4\eta^2 \\ -16\epsilon_{\rightarrow}{}^2(2\varsigma^3\epsilon_{\leftarrow}^3 - 14\varsigma\epsilon_{\leftarrow}\epsilon_{\rightarrow}{}^2)\eta - 16\epsilon_{\rightarrow}{}^2\left(\epsilon_{\rightarrow}{}^4 + 2\varsigma^2\epsilon_{\leftarrow}{}^2\epsilon_{\rightarrow}{}^2 + \varsigma^4\epsilon_{\leftarrow}{}^4\right). \end{gathered} \tag{S7.22}$$

To form the steady state (real eigenvalues), it requires $\Delta > 0$. Thus, the nonlinear threshold g_s can be obtained by solving $\Delta = 0$, i.e.,

$$\begin{gathered} \eta^4 + 2\varsigma\epsilon_{\leftarrow}\eta^3 - 11\epsilon_{\rightarrow}{}^2\eta^2 \\ -(2\varsigma^3\epsilon_{\leftarrow}^3 - 14\varsigma\epsilon_{\leftarrow}\epsilon_{\rightarrow}{}^2)\eta - \left(\epsilon_{\rightarrow}{}^4 + 2\varsigma^2\epsilon_{\leftarrow}{}^2\epsilon_{\rightarrow}{}^2 + \varsigma^4\epsilon_{\leftarrow}{}^4\right) = 0. \end{gathered} \tag{S7.23}$$

Solving the above equation fully can be performed numerically. To reveal the underlying mechanism, we consider $\gamma \to 1$, where $\varsigma \to 0$, Eq. (S7.23) can be simplified to

$$\eta^4 - 11\epsilon_{\rightarrow}{}^2\eta^2 - \epsilon_{\rightarrow}{}^4 = 0. \tag{S7.24}$$

Solving Eq. (S7.24) yields

$$\eta = \pm\sqrt{\frac{11 + 5\sqrt{5}}{2}}\epsilon_{\rightarrow} \Rightarrow g_s = \sqrt{\frac{11 + 5\sqrt{5}}{2}}\frac{\epsilon_{\rightarrow}}{W}. \tag{S7.25}$$

This gives the analytical form of g_s. Figure S11(a) shows g_s as a function of γ obtained numerically (green line) and analytically (blue line). Despite deviations between the two, the same trend unveils that g_s is competing with $\epsilon_{\rightarrow}$, which determines wave dispersion or broadening at the skin-mode localization sites.

Similarly, for g_n, considering the expansion coefficients $c_{\boldsymbol{R}}$ being nonzero at $c_{(0,0)}$ and $c_{(-1,0)}$ [see Fig. 3(b)], Eq. (S7.14) can be approximated as

$$Ec_{(0,0)} = gWc_{(0,0)}^3 + \epsilon_{(0,0),(0,0)}c_{(0,0)} + \epsilon_{(0,0),(-1,0)}c_{(-1,0)}, \tag{S7.26}$$

$$Ec_{(-1,0)} = gWc_{(-1,0)}^3 + \epsilon_{(-1,0),(-1,0)}c_{(-1,0)} + \epsilon_{(-1,0),(0,0)}c_{(0,0)}. \tag{S7.27}$$

Using a similar derivation as discussed above, we end up with (considering $\gamma \to 1$)

$$g_n = \sqrt{\frac{11+5\sqrt{5}}{2}\frac{\epsilon_{\leftarrow}}{W}}. \tag{S7.28}$$

This gives the analytical form of g_n. Figure S11(b) shows g_n as a function of γ obtained numerically (red line) and analytically (blue line). Although there are deviations, Fig. S11(b) unveils that g_n is competing with $\epsilon_{\leftarrow}$, which determines wave dispersion or broadening at sites where skin modes are not localized. This is in sharp contrast to g_s, revealing distinct formation mechanisms for SMASs and NRDSs.

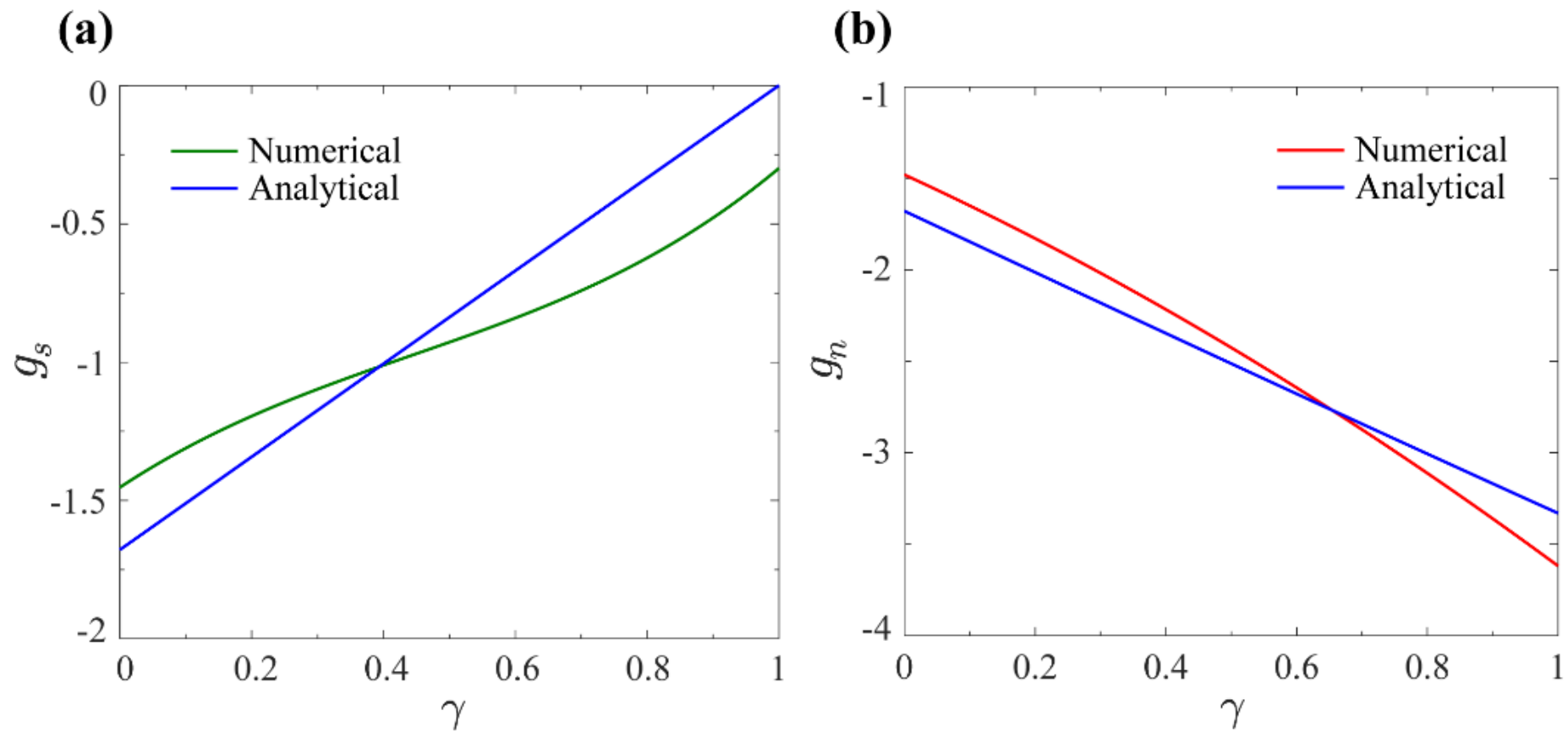

Figure S11. (a,b) Comparison of g_s (a) and g_n (b) versus γ between numerical [reproduced from the green and red lines in Fig. 3(a)] and analytical [Eqs. (S7.25) and (S7.28)] results.

Section VIII. Impact of the y direction on soliton formation

In this section, we examine the impact of the y direction on soliton formation. As displayed in Fig. 4 in the main text, we have tuned the interchain hoppings t_y along y to smoothly transition from one dimension to two dimensions, and then find that soliton thresholds are determined by NHSE, which is in sharp contrast to the crossover of soliton thresholds in Hermitian lattices when changing dimensionality.

To illustrate, we display soliton thresholds as a function of soliton emergence positions (n_x, n_y) for various γ and t_y in Fig. S12. Obviously, $t_y = 0$ is the one-dimensional case, and as γ increases, i.e., Figs. S12(a)-(d), the threshold profile changes dramatically. Such a dramatic change also occurs when $t_y = 0.5\kappa$, which corresponds to a two-dimensional case, as shown in Figs. S12(e)-(h). Although the threshold profile differs in the one- and two-dimensional Hermitian case [Figs. S12(a) and S12(e)], it resembles when γ is stronger, demonstrating the dominant role of the NHSE.

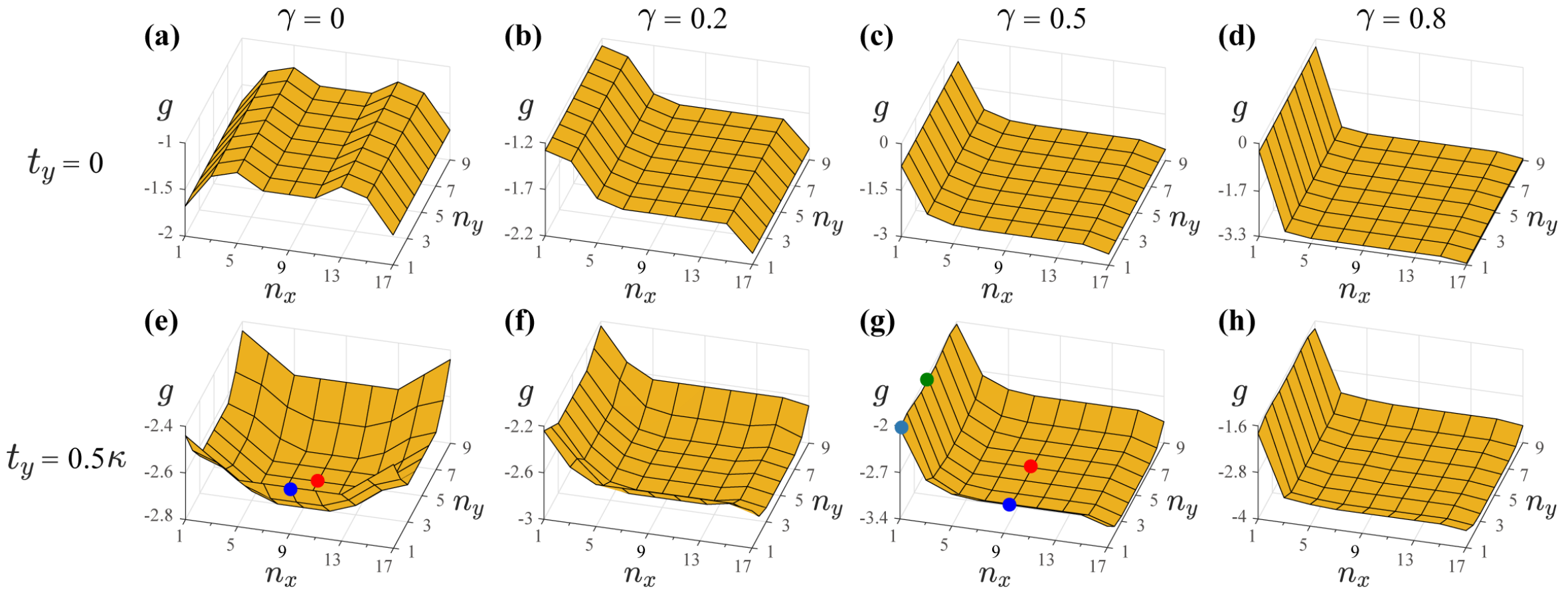

Figure S12. Thresholds for solitons localized at different lattice positions of the stacked nonreciprocal SSH model. (a)-(d) [(e)-(h)] correspond to $t_y = 0$ ($t_y = 0.5\kappa$) when $\gamma = 0$, $\gamma = 0.2$, $\gamma = 0.5$, and $\gamma = 0.8$. The colored dots mark several soliton thresholds for demonstration in Fig. S13. We set $N_x = N_y = 9$ here, and other parameters are identical to those in Fig. 2 of the main text.

From Fig. S12, it is not hard to expect that soliton profiles shall highly depend on y when we investigate solitons at distinct y positions. Figure S13 displays soliton profiles at the lattice positions marked in Figs. S12(e) and S12(g). Clearly, solitons located at different y coordinates do have distinct profiles, both for the Hermitian [Fig. S13(a)] and non-Hermitian [Fig. S13(b)] cases. Therefore, similar features of the soliton in Figs. 2(d) and 2(e) of the main text are from the same y-coordinate chosen to exhibit.

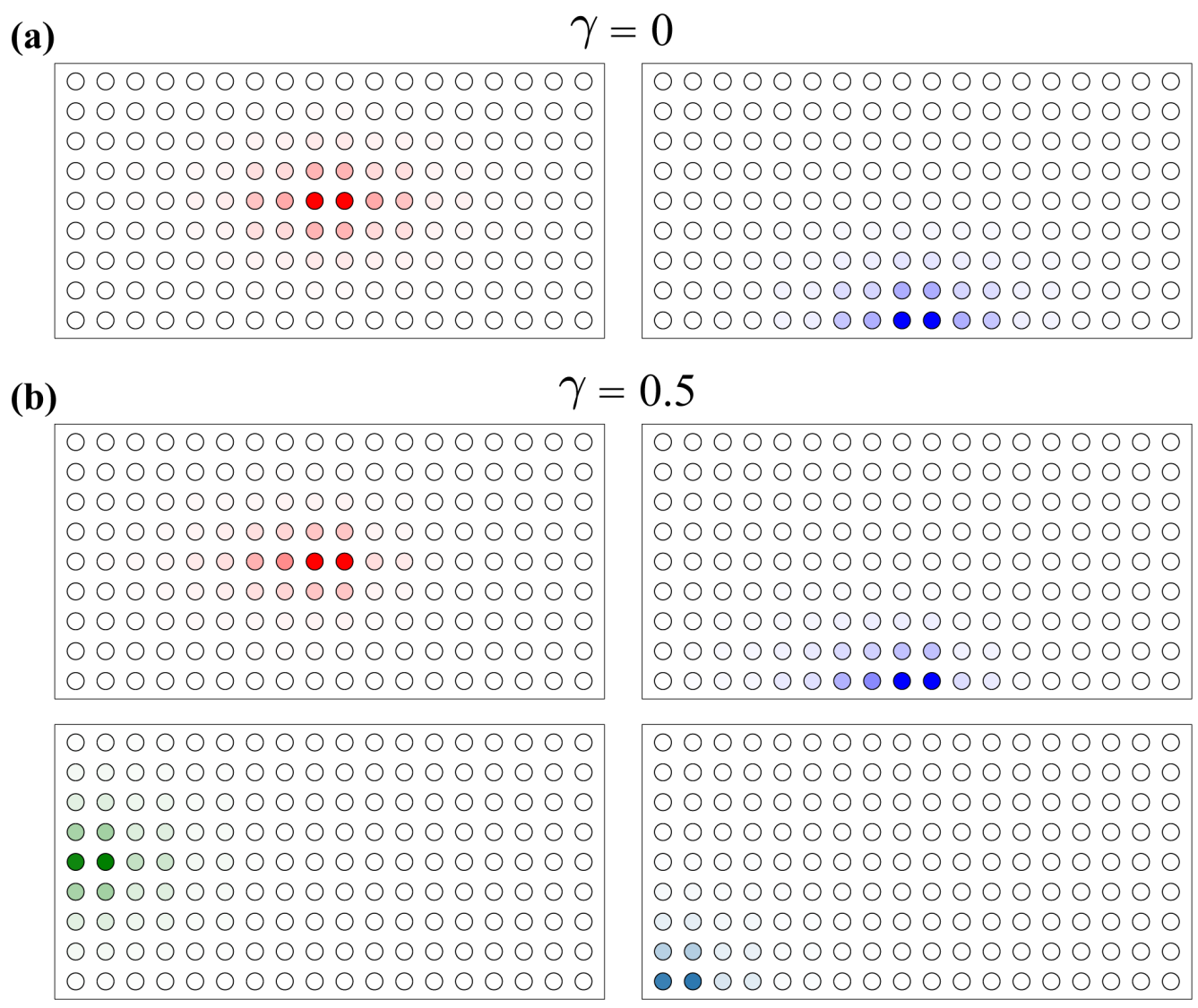

Figure S13. $|\psi_n|$ for solitons localized at different positions for $\gamma = 0$ (a) and $\gamma = 0.5$ (b). These solitons correspond to the colored dots highlighted in Figs. S12(e) and S12(g). The color shading represents the amplitude $|\psi_n|$.

Section IX. Dynamical accessibility of solitons

In this section, we investigate the dynamical accessibility of SMASs and NRDSs by selectively exciting two lattice sites in their localized regions and observing the resulting dynamics. Specifically, we excite sites $(n_x, n_y) = (1,3)$ and $(2,3)$ as edge excitation for SMASs, and sites $(9,3)$ and $(10,3)$ as bulk excitation for NRDSs. Under edge excitation, solitons are absent for $|g| < |g_s|$, whereas clear soliton features emerge when $|g| > |g_s|$ [Figs. S14(a) and S14(b)].

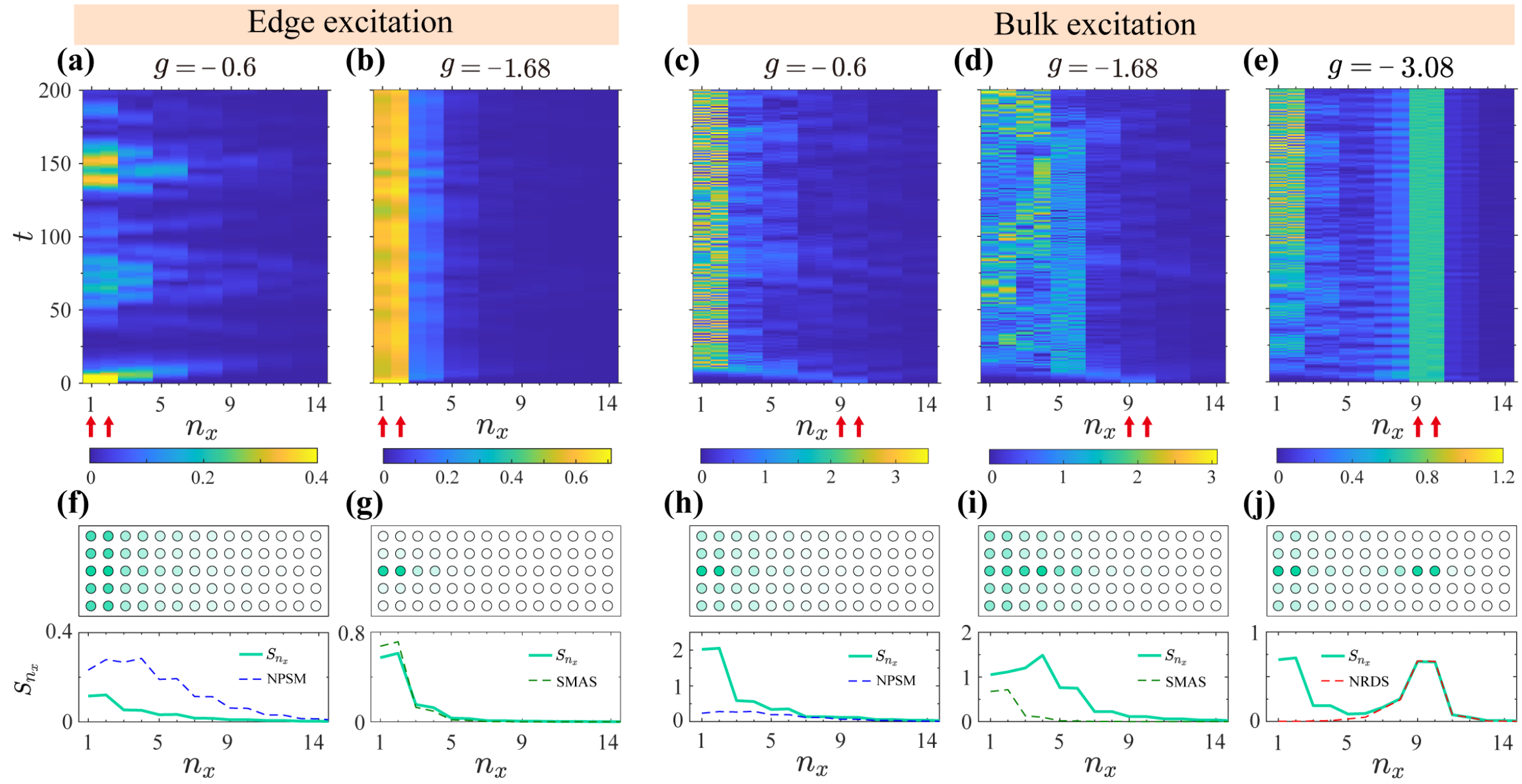

Figure S14. (a,b) Time evolution of $|\Psi_{(n_x,n_y=3)}(t)|$ for a skin mode (a) and an SMAS (b) under edge excitation. We use the two-site excitation at the favorable boundary, i.e., $\Psi_{\boldsymbol{n}}(0) = \frac{\sqrt{2}}{2}\delta_{n_x,1}\delta_{n_y,3} + \frac{\sqrt{2}}{2}\delta_{n_x,2}\delta_{n_y,3}$, illustrated by red arrows. (c)-(e) Time evolution of $|\Psi_{(n_x,n_y=3)}(t)|$ for various nonlinearities g under bulk excitation. The excitation is selected at the center of the lattice, i.e., $\Psi_{\boldsymbol{n}}(0) = \frac{\sqrt{2}}{2}\delta_{n_x,9}\delta_{n_y,3} + \frac{\sqrt{2}}{2}\delta_{n_x,10}\delta_{n_y,3}$, illustrated by red arrows. (f-j) Spatial distribution of the time-averaged wavefunction amplitude $S_{\boldsymbol{n}}$ (upper panels) corresponding to (a-e), where color shading represents the magnitude. The lower panels compare $S_{(n_x,n_y=3)}$ (solid lines) with $|\psi_{(n_x,n_y=3)}|$ of the nonlinear eigenstates (dashed lines). Their corresponding values of g are labelled in the panels, and other parameters follow those in Fig. 2 in the main text.

To quantify, we compute the time-averaged wavefunction amplitude

$$S_{\boldsymbol{n}} = \frac{1}{T/2}\int_{T/2}^{T} |\Psi_{\boldsymbol{n}}(t)| \mathrm{d}t\,, \tag{S9.1}$$

where T is the total recorded evolution time [10]. The upper and lower panels of Figs. S14(f) and S14(g) display the distribution of $S_{\boldsymbol{n}}$ across the entire lattice and along the slice $n_y = 3$, respectively. By comparing the dynamical result with the nonlinear eigenstate [Fig. S14(g), solid and dashed lines], we find that SMASs are clearly resolved, thereby confirming their accessibility via edge excitation.

Under bulk excitation and for nonlinearity below the threshold g_n, no solitons are observed. In contrast, wavefunctions accumulate toward the skin boundary [Figs. S14(c) and S14(d)], indicating that SMASs cannot be distinguished in this case. However, we observe the formation of solitons localized at the excitation sites when $|g| > |g_n|$ [Fig. S14(e)]. The upper and lower panels of Figs. S14(h) to S14(j) display the distribution of $S_{\boldsymbol{n}}$ across the entire lattice and along the slice $n_y = 3$, respectively. By comparing them with the nonlinear eigenstates (solid and dashed lines), we have further verified our findings above: that bulk excitation can resolve NRDSs but not SMASs. The NRDSs here encompass both the trap-skin states and the shifted-trap-skin states, as defined in Ref. [10], due to the formation mechanism of solitons.

References

1. Gong Z, Ashida Y and Kawabata K *et al*. Topological phases of non-Hermitian systems. *Phys Rev X* 2018; **8**: 031079.
2. Kawabata K, Shiozaki K and Ueda M *et al*. Symmetry and topology in non-Hermitian physics. *Phys Rev X* 2019; **9**: 041015.
3. Yao S and Wang Z. Edge states and topological invariants of non-Hermitian systems. *Phys Rev Lett* 2018; **121**: 086803.
4. Schindler F, Bulchandani VB and Benalcazar WA. Nonlinear breathers with crystalline symmetries. *Phys Rev B* 2025; **111**: 064312.
5. Manda BM, Carretero-González R and Kevrekidis PG *et al*. Skin modes in a nonlinear Hatano-Nelson model. *Phys Rev B* 2024; **109**: 094308.
6. Kapitula T and Promislow K. Spectral and dynamical stability of nonlinear waves. *Springer, New York* 2013.
7. Alfimov GL, Kevrekidis PG and Konotop VV *et al*. Wannier functions analysis of the nonlinear Schrödinger equation with a periodic potential. *Phys Rev E* 2002; **66**: 046608.
8. Jürgensen M and Rechtsman MC. Chern number governs soliton motion in nonlinear Thouless pumps. *Phys Rev Lett* 2022; **128**: 113901.
9. Fu Q, Wang P and Kartashov YV *et al*. Nonlinear Thouless pumping: solitons and transport breakdown. *Phys Rev Lett* 2022; **128**: 154101.
10. Ezawa M. Dynamical nonlinear higher-order non-Hermitian skin effects and topological trap-skin phase. *Phys Rev B* 2022; **105**: 125421.
